# From degrader to producer: reversing the gallic acid metabolism of *Pseudomonas putida* KT2440

**DOI:** 10.1007/s10123-022-00282-5

**Published:** 2022-11-11

**Authors:** Felipe M. S. Dias, Raoní K. Pantoja, José Gregório C. Gomez, Luiziana F. Silva

**Affiliations:** 1 Department of Microbiology, Institute of Biomedical Sciences, University of São Paulo, São Paulo, 05508-900 Brazil; 2 Centre for Genomic Regulation (CRG), The Barcelona Institute of Science and Technology, Dr. Aiguader 88, Barcelona, 08003 Spain; 3 Universitat Pompeu Fabra (UPF), Barcelona, Spain

**Keywords:** Aromatic compounds, Gallic acid, Metabolic engineering, Microbial cell factory, *Pseudomonas putida* KT2440, Synthetic biology

## Abstract

Gallic acid is a powerful antioxidant with multiple therapeutic applications, usually obtained from the acidic hydrolysis of tannins produced by many plants. As this process generates a considerable amount of toxic waste, the use of tannases or tannase-producing microorganisms has become a greener alternative over the last years. However, their high costs still impose some barriers for industrial scalability, requiring solutions that could be both greener and cost-effective. Since *Pseudomonas putida* KT2440 is a powerful degrader of gallic acid, its metabolism offers pathways that can be engineered to produce it from cheap and renewable carbon sources, such as the crude glycerol generated in biodiesel units. In this study, a synthetic operon with the heterologous genes *aro*G4, *qui*C and *pob*A* was developed and expressed in *P. putida*, based on an *in silico* analysis of possible metabolic routes, resulting in no production. Then, the sequences *pca*HG and *gal*TAPR were deleted from the genome of this strain to avoid the degradation of gallic acid and its main intermediate, the protocatechuic acid. This mutant was transformed with the vector containing the synthetic operon and was finally able to convert glycerol into gallic acid. Production assays in shaker showed a final concentration of 346.7 ± 0.004 mg L^−1^ gallic acid after 72 h.

**Supplementary Information:**

The online version contains supplementary material available at 10.1007/s10123-022-00282-5.

## Introduction

The role of oxidative stress in the pathophysiology of chronic conditions such as cancer, atherosclerosis and diabetes has been extensively described for several years (García-Sánchez et al. 2020). Due to its high importance, many studies have tested different mechanisms to treat it in order to reduce the level of severity of these diseases, pointing out the use of natural antioxidants as promising molecules, both for pharmacological treatments and prevention through supplementation (Sharifi-Rad et al. 2020). Among them, gallic acid (GA) and its derivatives have figured as strong agents against oxidative stress (Gao et al. 2019; Bai et al. 2021) and as a potential alternative to fight antibiotic-resistant bacteria (Kosuru et al. 2018). Furthermore, recent discoveries have also indicated that these molecules can be potentially explored to reduce high levels of oxidative stress in patients with strong COVID-19 (coronavirus disease) reactions (Diniz et al. 2020) and inhibit the activity of some coronavirus non-structural proteins (Umar et al. 2021).

The natural biosynthesis of this molecule, also known as 3,4,5-trihydroxybenzoic acid, happens in many plants as part of the Shikimate Pathway, the metabolic pathway responsible for the production of aromatic amino acids (tyrosine, phenylalanine and tryptophan) and also present in bacteria (Muir et al. 2011). Through this pathway, some plants, such as sumac (*Rhus coriaria*), accumulate polymers known as tannic acids that can be extracted and hydrolyzed into GA and other molecules. Nevertheless, in spite of being a non-expensive process, it generates as a byproduct 6.5 m^3^ of liquid toxic waste for every 1 ton of GA produced (Wu et al. 2017). As an alternative, the use of tannases or tannase-producing microorganisms, such as yeast, fungi and bacteria has been tested in many studies (Dhiman et al. 2018; Dhiman and Mukherjee 2020); however, the toxicity of tannic acid to these microorganisms and low hydrolysis rates by isolated tannases still make these options less attractive than the prior in terms of cost-effectiveness (Dhiman et al. 2018).

Taking that into consideration, a new biotechnological approach has become a promising trend: the de novo production and release of aromatic compounds by recombinant bacteria (Huccetogullari et al. 2019). By using residues from biofuel plants such as starch or crude glycerol as non-expensive feedstocks, the cultivation of these microorganisms can reduce even more the environmental impacts of these facilities and add high-value molecules to their portfolio, allowing the development of new biorefinery models (Dias et al. 2017; Braga and Faria 2022). Many of these bacteria, normally designated as “SynBio chassis” (de Lorenzo et al. 2021), have already been modified in order to do these bioprocesses in a safe and effective industrial scale, *Escherichia coli* being the most commonly used (Dias et al. 2017; Braga and Faria 2022).

Even though the *de novo* production of GA has been already reported in recombinant *E. coli* (Kambourakis et al. 2000; Muir et al. 2011; Moriwaki et al. 2019), this bioprocess requires an organism with a more robust metabolism, capable to provide a considerable reducing power to the synthetic route. In that case, an alternative SynBio chassis with great potential to be explored for this purpose is *Pseudomonas putida* (Molina-Santiago et al. 2016; Schwanemann et al. 2020; Weimer et al. 2020). Besides its safety and relatively fast growth in bioreactors, this species has also a very versatile metabolism with diverse degradation routes of aromatic compounds (Khmelenina et al. 2019) and a complex glucose metabolism that favors the accumulation of reductive power in the form of NADPH (Nikel et al. 2015a). Furthermore, *P. putida* is able to consume the crude glycerol generated as a residue in biodiesel plants, which normally contains many compounds that inhibit the growth of many bacteria in bioreactors (Verhoef et al. 2014), and has also several tools for genome editing and expression of synthetic circuits, making it easy to be engineered (Silva-Rocha et al. 2013; Weimer et al. 2020).

In this study, *de novo* production of GA from glycerol by a mutant and recombinant strain of *P. putida* KT2440 was described for the first time, opening new opportunities for the industrial use of this species in the production of high-value aromatic compounds.

## Materials and methods

### Strains, genes and culture media

All strains used in the present study are listed in Table 1. The host strain referred here as parental strain (P) already had a deletion of the gene *glp*R (Nikel et al. 2015b).

**Table 1:** Strains and plasmids used in this project

Bacterial strains or plasmids	Relevant characteristics	Reference or source
Strains		
*Escherichia coli*		
K12 MG1655	F- lambda- ilvG- rfb-50 rph-1	(Tyo et al. 2009)
DH10B	Cloning host; F- mcrA Δ(mrr-hsdRMS-mcrBC) Φ80dlacZΔM15 ΔlacX74 endA1 recA1 deoR Δ(ara,leu)7697 araD139 galU galK nupG rpsL λ-	(Durfee et al. 2008)
S17-1 λ pir	Helper strain for conjugation; TpR SmR recA, thi, pro, hsdR-M + RP4: 2-Tc:Mu: Km Tn7 λpir	(Simon et al. 1983)
*Pseudomonas putida*		
P	Parental strain of *P. putida* KT2440 rmo mod + glpr- (DSM 6125)	(Nikel et al. 2015b)
P_J0	Parental strain (P) with plasmid J0	This work
P_JQP	Parental strain (P) with plasmid JQP	This work
P_JAQP	Parental strain (P) with plasmid JAQP	This work
Gal1	Mutant strain: Parental strain (P) with deletions Δ*gal*TAPR and Δ*pca*HG	This work
Gal1_J0	Mutant strain Gal1 with plasmid J0	This work
Gal1_JQP	Mutant strain Gal1 with plasmid JQP	This work
Gal1_JAQP	Mutant strain Gal1 with plasmid JAQP	This work
Plasmids		
J0	Expression plasmid pJN105 without any heterologous genes; araC-P_BAD_ cassette cloned in pBBR1MCS-5, *cb*R/*amp*R	(Newman and Fuqua 1999)
JQ	Expression plasmid pJN105 containing the gene *qui*C from *A. baumannii* ATCC 19606	This work
JQP	Expression plasmid pJN105 containing the genes *qui*C and *pob*A* (*pob*A from *P. aeruginosa* ATCC 9027 with mutation Tyr385Phe)	This work
JAQP	Expression plasmidpJN105 containing genes *qui*C, *pob*A* and *aro*G4 (*aro*G from *E. coli* K12 MG1655 with mutation Pro150Leu)	This work
pEX-18	Helper suicide plasmid used for conjugation and recombination; oriT + sacB + , gene replacement vector with MCS from pUC18. *gm*R	(Hoang et al. 1998)
pEX-18::*galTAPR cassette*	pEX-18 with upstream and downstream homology sequences forming a cassette for homologous recombination to delete *gal*TAPR	This work
pEX-18::*pcaHA cassette*	pEX-18 with upstream and downstream homology sequences forming a cassette for homologous recombination to delete *pca*HG	This work

Lysogeny Broth (LB) culture medium, rich in nutrients was used to grow all strains, containing the following composition (g L^−1^): tryptone (10), yeast extract (5), NaCl (5) and agar (20), the latter being added only in solid media. In addition, liquid Mineral Medium (MM) was used for the production of GA, having the following composition (g L^−1^): Na_2_HPO_4_ (3.5), KH_2_PO_4_ (1.5), (NH_4_)_2_SO_4_ (1), MgSO_4_.7H_2_O (0.2), CaCl_2_.2H_2_O (0.01), ammonium iron (III) citrate (0.06) and trace element solution (1 ml L^−1^). The trace element solution contained (g/L): H_3_BO_3_ (0.30) CoCl_2_.6H_2_O (0.20), ZnSO_4_.7H_2_O (0.10), MnCl_2_.4H_2_O (0.03), NaMoO_4_.2H_2_O (0.03), NiCl_2_.6H_2_O (0.02) and CuSO_4_.5H_2_O (0.01), adapted from Ramsay et al. (1990). In MM, pure glycerol (85%, Merck) was added at a final concentration of 10 g L^−1^. For the preparation of the Cetrimide Agar medium (solid), cetrimide agar (46.7 g L^−1^) was added in a solution with 10% glycerol (volume volume^−1^).

In addition, carbenicillin was added to the medium when the cells had the plasmid pEX18 (final concentration of 200 µg mL^−1^ for *P. putida* and 150 µg mL^−1^ for *E. coli*), which contains the *cb*R/*amp*R gene that confers resistance to this antibiotic; and gentamicin was added when the bacterium had the plasmid pJN105 (final concentration of 10 µg mL^−1^ for *P. putida* and 40 µg mL^−1^ for *E. coli*), which contains the *gm*R gene that confers resistance to this antibiotic.

### *In silico* analysis

**Fig. 1:**
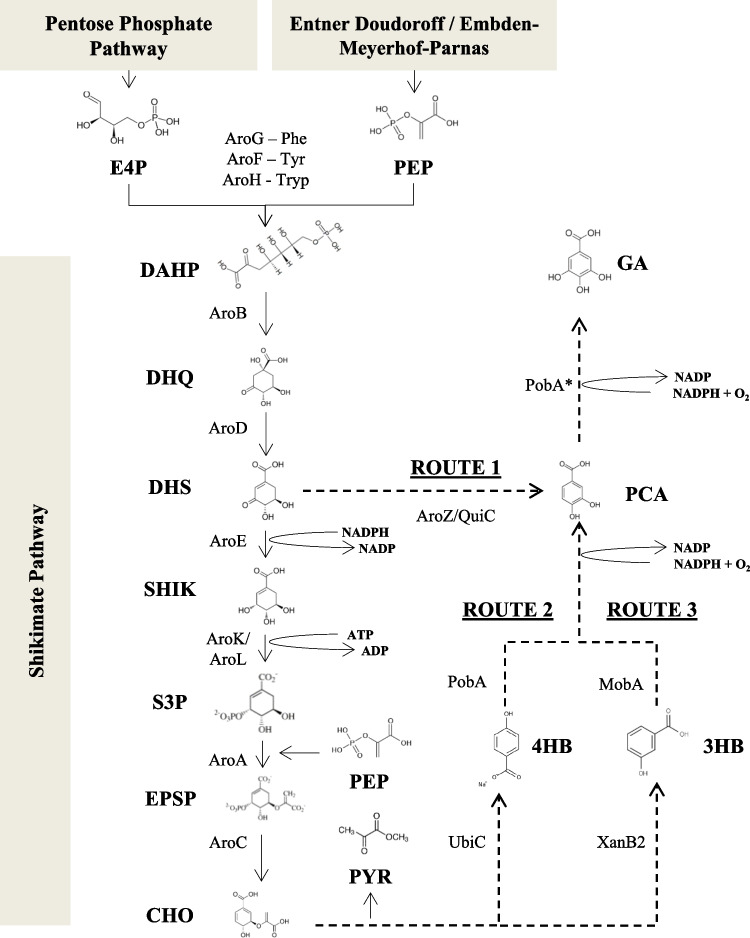
Metabolic routes that could be expressed in *P. putida* for the production of GA (dashed arrows) with heterologous genes, all depending on the availability of intermediates from the Shikimate Pathway of *P. putida* KT2440 metabolism (full arrows). The metabolites and other molecules are: 3HB, 3-Hydroxybenzoate; 4HB, 4-Hydroxybenzoate; ADP, Adenosine diphosphate; ATP, Adenosine triphosphate; CHO, Chorismate; DHAP, 3-Deoxy-D-arabino-heptulosonate 7-phosphate; DHQ, 3-Dehydroquinate; DHS, 3-Dehydroshikimate; E4P, Eryhtrose-4-Phosphate; EPSP, 5-enolpyruvylshikimate-3-phosphate; GA, Gallic acid; NADP, Nicotinamide adenine dinucleotide phosphate; NADPH, Reduced form of NADP; O_2_, Oxygen; PCA, Protocatechuate; PEP, Phosphoenolpyruvate; PYR, Pyruvate; S3P, Shikimate-3-Phosphate; SHI, Shikimate. The enzymes are: AroA, 3-phosphoshikimate 1-carboxyvinyltransferase; AroB, 3-dehydroquinate synthase; AroC, Chorismate synthase; AroD, 3-dehydroquinate dehydratase; AroE, Shikimate dehydrogenase; AroF, AroG and AroH: Phospho-2-dehydro-3-deoxyheptonate aldolases inhibited by tyrosine, phenylalanine and tryptophan, respectively; AroK/AroL, Shikimate kinases; AroZ, 3-dehydroshikimate dehydratase of *Klebsiella baumannii*; MobA, 3-hydroxybenzoate 4-monooxygenase of *Comamonas testosterone*; PobA and PobA*, p-hydroxybenzoate hydroxylase of *Pseudomonas aeruginosa* and its mutated version (Tyr385Phe), respectively; QuiC, 3-dehydroshikimate dehydratase of *Acinetobacter baumannii*; UbiC, Chorismate pyruvate lyase of *Escherichia coli*; XanB2, Chorismatase of *Xanthomonas campestris* pv. *campestri*

Stoichiometry-based pathway search tools offer a powerful support to understand metabolic fluxes in a cell, providing a starting point for Metabolic Engineering initiatives (Trinh et al. 2009; Wang et al. 2017). Once knowing the possible reactions along with the internal and external metabolites behind the bioproduction of a target molecule, it is possible to list all groups of non-redundant reactions that keep its production in the steady-state, the so-called elementary modes (Trinh et al. 2009). Based on the literature (Kambourakis et al. 2000) (Frost 2000) and an extensive search on the Kyoto Encyclopedia of Genes and Genomes database (KEEG) (www.genome.jp/kegg/kegg2), three routes from which GA could be produced from the Shikimate Pathway in *P. putida* were identified (Fig. 1).

Adopting the premise that the production of GA by recombinant bacteria should occur without growth since the Shikimate Pathway would consume precursors used in the production of biomass (eryhtrose-4-phosphate — E4P and phosphoenolpyruvate — PEP) also resulting in no generation of aromatic amino acids (phenylalanine, tyrosine and tryptophan), all chemical reactions necessary for the conversion of glycerol or glucose into GA and the maintenance of cellular respiration were mapped (Nikel et al. 2015a, 2015b; Poblete-Castro et al. 2020). The three synthetic routes (Fig. 1) were also included in the model as alternatives for GA production along with the reactions promoted by the transhydrogenases present in *P. putida*, which enable the conversion of NADH to NADPH and vice versa (Nikel et al. 2016). Then, these reactions were coded in two separate input files, one considering glycerol and the other glucose as sole carbon sources (Tools S1 and S2, respectively). Both were submitted to an *in silico* analysis using Metatool (pinguin.biologie.uni-jena.de/bioinformatik/networks/metatool/metatool5.1/metatool5.1.html), which generated one output file each, with the details of all possible elementary modes (Tables S1 and S2).

The elementary modes with the highest yields (g g^−1^) were identified for both carbon sources (Table 1 and Table S3), allowing the definition of which synthetic metabolic route should be included in the metabolic network with the aid of expression vectors. In this model, the only cofactors considered were the pairs NAD/NADH, NADP/NADPH, FAD/FADH_2_ and ADP/ATP and some molecules, such as H_2_O were not included to simplify it (Tools S1 and S2).

### Genes and expression plasmids

In order to express Route 1 as the target metabolic pathway, the synthetic operon developed for *P. putida* needed to express genes responsible for the conversion of 3-dehydroshikimate (DHS) to protocatechuate (PCA) and then of PCA to GA (Fig. 1). According to a patent that claimed the bacterial production of muconic acid from PCA (Bui et al. 2013), the 3-dehydroshiquimate dehydratase (QuiC) found in *Acinetobacter baumannii* had demonstrated a superior performance compared to the orthologous versions of other organisms, such as the one from *K. baumannii* expressed in *E. coli* by Kambourakis et al. (2000) (Frost 2000) for the first reaction. However, the conversion of PCA to GA was only possible with a mutant version (Tyr385Phe) of the enzyme p-hydroxybenzoate hydroxylase (PobA*) from *Pseudomonas aeruginosa*, which hydroxylates PCA instead of 4-hydroxybenzoate (4HB) (Kambourakis et al. 2000; Muir et al. 2011; Moriwaki et al. 2019). These two reactions would be enough for the proposed operon; however, in many organisms the condensation of E4P and PEP in 3-deoxy-D-arabino-heptulosonate 7-phosfate (DAHP) is catalyzed by the enzyme phospho-2-dehydro-3-deoxyheptonate aldolase in different versions that can be inhibited by phenylalanine (AroG), tyrosine (AroF) or tryptophan (AroH). So, to avoid this inhibition, it was also necessary to express a mutant version of the gene *aro*G, called *aro*G4 (Pro150Leu), insensitive to phenylalanine (Doroshenko et al. 2010).

**Fig. 2:**
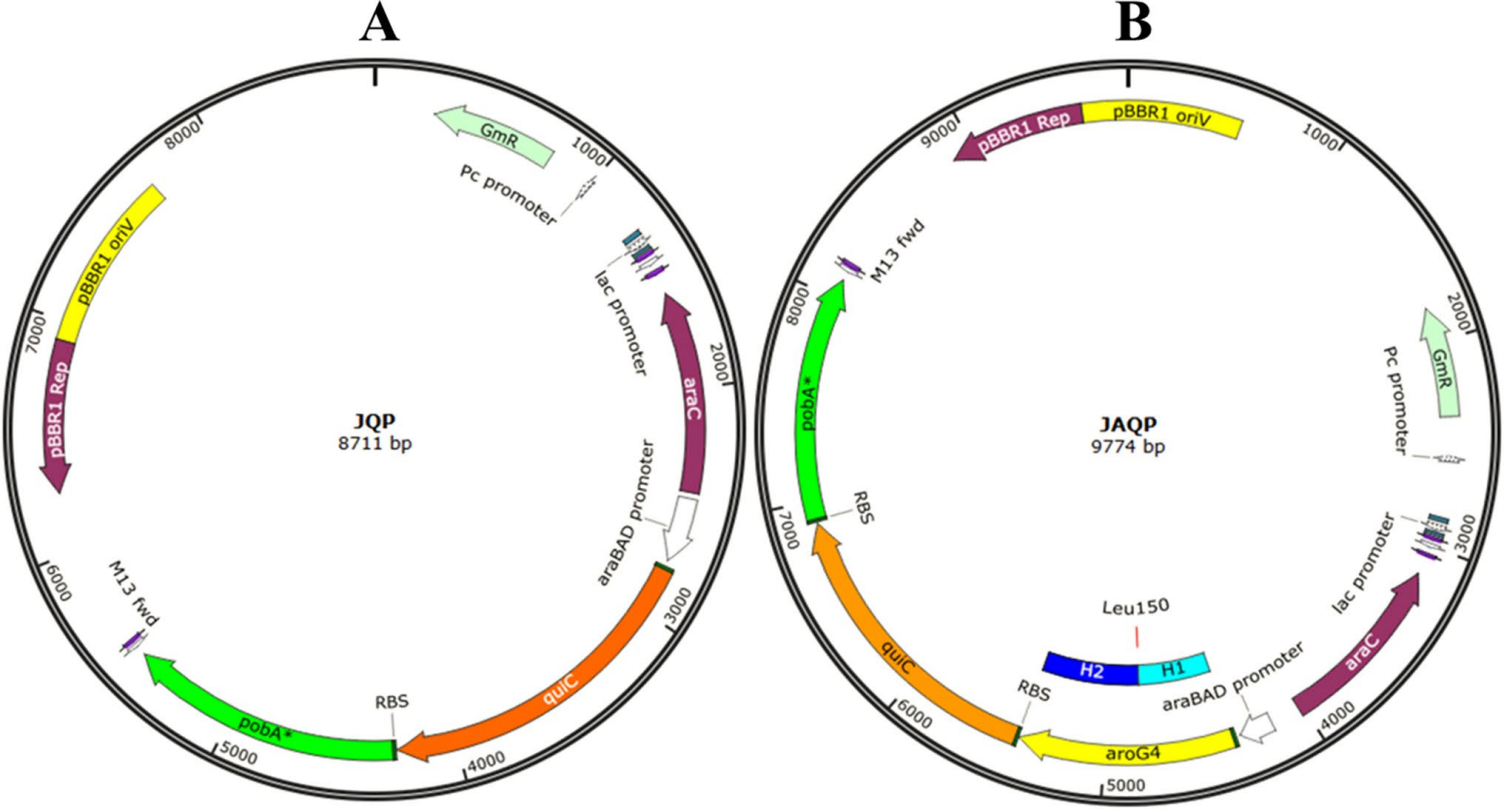
Maps of the expression plasmids JQP (A) and JAQP (B) done with SnapGene (www.snapgene.com/). Highlighted are the upstream and downstream homologous sequences constructed to clone the *aroG4* gene in the JQP plasmid (H1, in light blue and H2, in dark blue)

The gene *qui*C was obtained from genomic DNA of *A. baumannii* ATCC 19,606, *pob*A was obtained from genomic DNA of *P. aeruginosa* ATCC 9027 and *aro*G was obtained from genomic DNA of *E. coli* K12 MG1655, using Polymerase Chain Reaction (PCR) (Saiki et al. 1988). The genomic DNAs were obtained with the Wizard® Genomic DNA Purification Kit (Promega). All primers used to isolate these genes through PCR are in the Table S4. For the gene *qui*C, the leading strand primer was designed to contain a conserved *Pseudomonas* ribosomal binding site (RBS), used in vectors constructed by Silva-Rocha et al. (2013), along with an EcoRI restriction site, while its reverse primer received an SmaI restriction site. To isolate the *pob*A* gene from a genomic DNA containing its parental strain version, the leading strand primer had the same RBS and a SmaI restriction site, while the reverse primer had the nucleotide exchange (T—> A) that would lead to the desired mutation (Tyr385Phe), in addition to a BcuI restriction site. Thus, both genes were amplified and purified using the Wizard® SV Gel and PCR Clean-Up System (Promega), subjected to the corresponding restriction enzymes FastDigest (Thermo Fischer™) and then ligated to the expression vector pJN105 (named here as J0) (Newman and Fuqua 1999) with the aid of the enzyme *T4 DNA Ligase* (Thermo Fischer™), starting with the insertion of *qui*C to form the JQ plasmid and, then, *pob*A* to form the JQP plasmid (Fig. 2).

To clone *aro*G4, the One-Step Sequence-and-Ligase-Independent Cloning (SLIC) method was applied (Jeong et al. 2012), in which the *E. coli* K12 MG1655 *aro*G gene was isolated in the form of two fragments, thereby using four primers with ends that were homologous so they could anneal with each other and vector JQP, which was digested with the enzyme EcoRI. To create cohesive ends for that, the three fragments were submitted to the exonucleolytic action 3'-5 'of the enzyme *T4 DNA Polymerase* (New England Biolabs®). The result of this annealing was introduced in *E. coli* DH10B by thermal shock and the bacterium repair system itself made the necessary covalent bonds to joint all fragments in a final plasmid. Both the reverse primer of the first fragment of *aro*G, and the primer of the leading strand of the second fragment were designed with the point mutation (C—> T), so the *aro*G4 version (Pro150Leu) could be inserted in the plasmid JQP, resulting in the JAQP version (Fig. 2). The set of expression plasmids constructed is summarized on Table 1.

All genes were isolated by PCR, using the high-fidelity enzymes *Phusion*® *High-Fidelity DNA Polymerase* (NEB) or *Q5® High-Fidelity DNA Polymerase* (NEB). In addition, PCR tests conducted to verify the cloning process used the *GoTaq® Green Master Mix* kit (Promega). All amplifications were validated with electrophoresis in 0.7% agarose gel (Figure S1) and after each amplification and digestion all amplicons were purified with the Wizard® SV Gel and PCR Clean-Up System (Promega). Furthermore, all plasmids cloned in *E. coli* DH10B were purified with Wizard® Plus SV Minipreps DNA Purification System (Promega). All primers and maps were designed using the software SnapGene (www.snapgene.com) and NEB Builder (nebuilder.neb.com). Furthermore, plasmid JAQP was sequenced according to the Colson and Sanger technique (Sanger and Coulson 1975). All primers used for sequencing are in Table S4.

### Genome editing

*P. putida* KT2440 is endowed with catabolic operons that encode enzymes responsible for the degradation of GA (operon *gal*TAP) and PCA (operon *pca*HG) (Nogales et al. 2011, 2017). In the case of the *gal*TAP operon, the enzyme gallic acid dioxygenase (GalA) is responsible for the first stage on the GA degradation route, the *gal*T gene encodes for a GA transporter in the inner membrane of the cell and *gal*P encodes for a porin in the outer membrane. This operon is regulated by a transcription factor encoded by *gal*R, whose expression is proportional to the concentration of GA in the environment (Nogales et al. 2011). At the same time, the *pca*HG operon encodes two subunits of the 3,4-dioxygenase protocatechuate enzyme (Khmelenina et al. 2019).

The deletion required an homologous recombination that was done with the aid of the vector pEX-18 (Hoang et al. 1998) and a protocol that consisted of adding to the vector a recombination cassette formed by the two sequences that flank the regions to be deleted (upstream and downstream homologous sequences of approximately 1000 bp) (Hmelo et al. 2015; Huang and Wilks 2017).

Since the *gal*R, *gal*T and *gal*P genes present homologies with other regions of the *P. putida* KT2440 genome (pseudomonas.com), the upstream and downstream homologous sequences were defined to delimit the region to be deleted from *gal*R to *gal*T, once choosing any other region withing both genes to only delete *gal*A could result in recombination in other parts of the genome. Once this mutant was obtained, a second round was performed in order to delete the *pca*HG operon, resulting in a final strain called Gal1 (*Pseudomonas putida* KT2440 Δ*glp*R Δ*gal*TAPR Δ*pca*HG).

At the beginning of the procedure, SLIC was used to obtain the construction containing the recombination cassette and the resulting plasmid was inserted by electrotransformation into an *E. coli* S17-1 λ pir, which was capable of transferring it by conjugation to *P. putida* on solid LB medium. The conjugation facilitated the insertion of the linear vector into the *P. putida* genome by the homologous recombination of one of the upstream or downstream homologous sequences, providing resistance to carbenicillin. The colonies formed were then chosen randomly and validated by PCR.

Four colonies with the first recombination validated by PCR were individually inoculated in test tubes containing LB medium without antibiotics to allow the growth of cells where a second recombination in the other homology sequence could take place, thereby removing the vector and the region of interest. After 12 h of cultivation, the cultures were diluted 10^4^ times and then sown in LB *medium* containing 15% sucrose (mass volume^−1^), so that cells that still had the vector would die due to the *sac*B gene, which converts sucrose into a toxic compound to bacteria. Then, the colonies that grew in the medium underwent a new validation by PCR, in order to assess whether they no longer presented the same band present in the amplification of the genomic DNA of the parental strain (P) (Fig. S2).

Furthermore, the genomic DNA of both regions was amplified by PCR, purified and then sequenced according to the Colson and Sanger technique (Sanger and Coulson 1975). The primers used are on Table S4.

### Production assays

The strains used were: P_J0, P_JQP and P_JAQP for the first assay, in order to evaluate whether the expression of the heterologous genes QuiC, PobA* and AroG4 by the parental strain were enough for the production of GA; P_JAQP and Gal1_JAQP for the second assay, in order to assess whether the gene deletions were important to detect GA in the broth, and Gal1_J0, Gal1_JQP and Gal1_JAQP for the third assay, to verify whether the expression of QuiC and PobA* by the mutant strain was enough or whether the expression of AroG4 was also an important requirement. In all assays, the strains were grown in triplicate in a mineral medium containing pure glycerol (10 g L^−1^) at 30 °C, with rotation at 150 rpm, from an optical density (OD_600_) equal to 0.1. Samples were taken after inoculation (0 h), and then, after 24 h and 72 h. The following measurements were done: OD_600_, cell dry mass (g), pH and glycerol, GA and PCA concentrations (g L^−1^) in the supernatant, after centrifugation and filtration. Right after the 24 h sample was taken, induction was done for all strains by the addition of L-arabinose in the remaining culture, maintaining a final concentration of this inducer equal to 0.2% (mass volume^−1^). The induction was necessary so the heterologous genes added to the plasmids could be expressed for the production of GA, given that the expression vector used (pJN105) had an araBAD promoter (Fig. 2).

The supernatant samples were analyzed in High Performance Liquid Chromatography (HPLC), using the Dionex equipment Ultimate 3000 model, Aminex HPX 87 H column, temperature 40 °C, mobile phase 0.5 mL min^−1^ with 0.005 mM of H_2_SO_4_ to quantify glycerol. GA and PCA were quantified in the first two assays in HPLC, using a Dionex model Ultimate 3000 equipment, Column Zorbax Eclipse Plus C18 (4.6 × 150 mm, particle: 3.5 um), eluted at 90% of acidified water with 0.1% acetic acid and 10% acetonitrile from 0 to 6 min, at a temperature of 45 °C, flow rate of 1.0 mL min^−1^ and UV detector 280 nm (GA) and 254 nm (PCA). Each molecule had a different retention time, facilitating the detection and quantification by this method (Fig. S3). In this assay, to assess the intracellular content of GA and PCA, the cell pellet of each sample was resuspended in distilled water and subjected to sonication in Ultracleaner 1400 (Milleto) with 10 pulses of 15 s each.

To quantify GA and PCA in the third assay, the supernatant samples were analyzed in an Agilent 1100 series equipment (Agilent, Waldbronn, Germany), with the separation in a Phenomenex (Torrance, CA, USA) Aqua C18 column at a temperature of 25 °C, flow rate of 1.0 mL min^−1^ and UV detector 280 nm. The mobile phase consisted of 2% (volume volume^−1^) of acetic acid in water (eluent A) and 0.5% acetic acid in water with acetonitrile (50:50, volume.volume^−1^; eluent B) using the following program gradient: 10 to 15% B (10 min), 15% B isocratic (3 min), 15 to 25% B (7 min), 25 to 55% B (30 min), 55 to 100% B (1 min), 100% isocratic (5 min), from 100 to 10% B (0.1 min). Each molecule had a different retention time, facilitating the detection and quantification by this method (Fig. S4).

### Growth assay in crude glycerol

All production assays were done with pure glycerol in order to enable the calculation of their yields, given that crude glycerol might contain other carbon sources in its impurities. Thus, to assess whether the gene deletions affected the ability of Gal1 to grow in crude glycerol at a faster rate than *E. coli* K12 MG1655, another important strain used to convert crude glycerol into bioproducts, the growth curves of both strains cultivated with crude glycerol as the only carbon source were compared.

A colony of Gal1 was pre-inoculated and cultivated in LB for 12 h and then inoculated in MM containing 10 g L^−1^ of crude glycerol (89.94%) from the Research Center of Petrobrás (Cenpes) in Guamaré (Rio Grande do Norte, Brazil) as the sole carbon source, adjusting the initial optical density (OD_600_) to 0.1. Then, triplicates of 700 μL corresponding to each culture were distributed in a 24-well plate and this was taken to a Synergy H1 plate reader (Biotek, VE, USA) that performed DO_600_ readings every 15 min, for 44 h, 30 °C. The lag times of both cultures were estimated from the absorbance values, as described by Dalgaard and Koutsoumanis (2001).

## Results

### Selecting the metabolic pathway for GA biosynthesis

From the input provided to MetaTool to identify the route with better conversion of glycerol or glucose into GA in *P. putida* (Fig. 1 and Tools S1 and S2, respectively) 355 elementary modes were obtained (Tables S1 and S2). After calculating the maximum yield (g g^−1^) for each elementary mode, it was possible to note that the synthetic route established by Kambourakis et al. (2000) in *E. coli*, named here as Route 1 (Fig. 1) was the one with the highest maximum yield when compared to the other routes either when glycerol or glucose were used as the only carbon sources (Table 2).

**Table 2:** List of elementary modes containing the highest yields (g/g) per carbon source and synthetic route chosen

Carbon source	Synthetic Route for GA production	Elementary modes	Maximum yield (g/g)
Glycerol	Route 1	54	0.7745
Glycerol	Routes 2 or 3	4, 5	0.6522
Glucose	Route 1	76, 98	0.7636
Glucose	Routes 2 or 3	73, 74, 81, 82, 91, 92, 93, 94	0.6408

For more details of what reactions were selected by Metatool to define the elementary modes highlighted on Table 2 see Table S3.

### Production assays

**Fig. 3:**
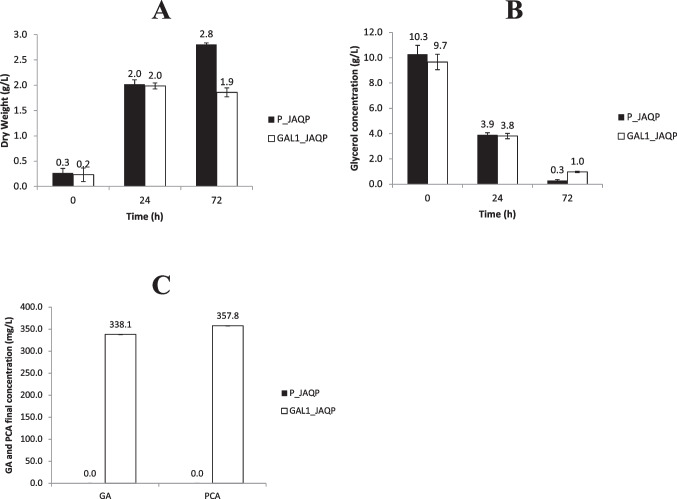
Measurements of cell dry weight (A) and glycerol concentration (B) over time, along with GA and PCA concentrations (C) after 72 h, comparing the double-mutant Gal1 (white) and the parental strain P (black), both transformed with plasmid JAQP

Three production assays were performed in shaker flasks. In the first assay, the parental strain (P) was transformed with plasmids J0 (control without any heterologous genes), JQP or JAQP, however, no GA or PCA were detected after a 72 h cultivation. Then, considering the hypothesis that this result was a consequence of the degradation of GA and PCA by the expression of the corresponding gene clusters in *P. putida*, a second assay was made, comparing the mutant strain Gal1 to the parental strain, both transformed with plasmid JAQP (Fig. 3). In the end of this assay, it was already possible to see that the culture medium of the flasks containing the strain Gal1_JAQP became darker (Fig. S5), probably due to oxidation reactions mediated by GA.

**Fig. 4:**
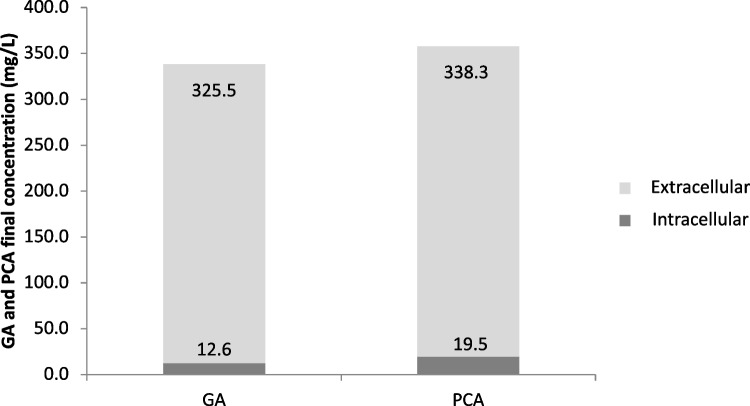
Intracellular (dark grey) and extracellular (light grey) quantification of GA and PCA after a 72 h assay with Gal1 containing the plasmid JAQP

As expected, the results showed that the double-mutant Gal1 was able to produce and release GA in the broth (Fig. S3), highlighting the importance of the deletions to guarantee that *P. putida* will no longer degrade it. Measurements of total (intra and extracellular concentrations) GA and PCA in Gal1_JAQP revealed that there was little presence of these compounds intracellularly, indicating that, for both molecules, secretion to the medium was high (Fig. 4).

**Fig. 5:**
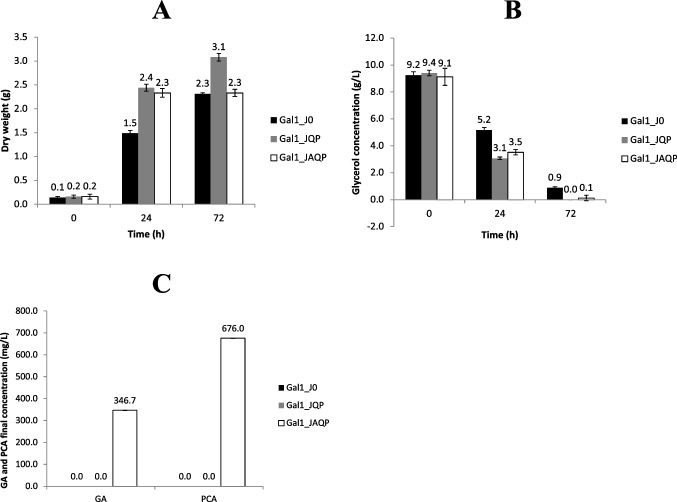
Measurements of cell dry weight (A) and glycerol concentrations (B) over time, along with GA and PCA concentration (C) after 72 h, comparing different strains of the double-mutant strain Gal1 transformed with plasmids J0 (black), JQP (grey) and JAQP (white)

In the third assay, the comparison of the double-mutant strain (Gal1) containing the plasmids J0, JQP or JAQP showed that the inclusion of *aro*G4 was necessary, with GA being detected only when this gene was expressed (Figs. 5 and S4).

### Growth assay in crude glycerol

**Fig. 6:**
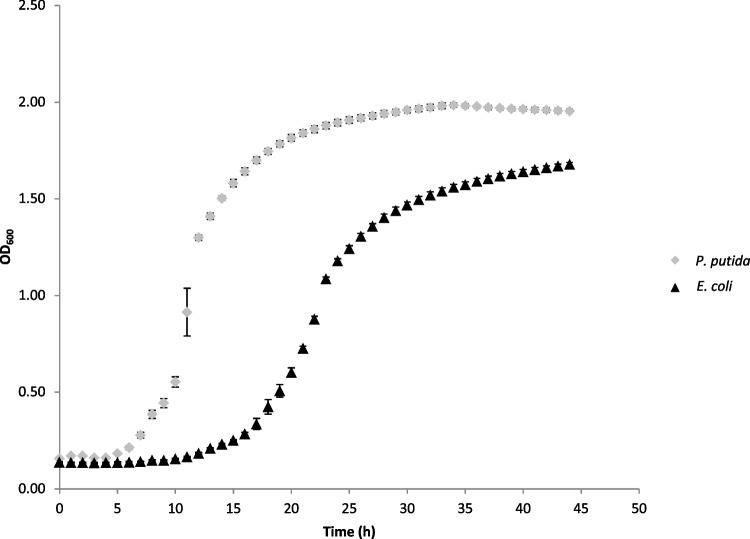
OD_600_ growth curves of *P. putida* Gal1 (grey diamonds) and *E. coli* K12 MG1655 (black triangles) cultivated in a 24-well plate with mineral medium containing crude glycerol as the only carbon source

The optical density (OD_600_) curves (Fig. 6) not only showed that the lag time was shorter for Gal1, reinforcing the importance of the Δ*glp*R mutation of the parental strain used (Nikel et al. 2015b), but also that Gal1 was able to reach a higher maximum OD_600_ before *E. coli*.

## Discussion

*P. putida* is a SynBio chassis endowed with a very versatile metabolism that allows it to degrade several aromatic compounds from the environment (Khmelenina et al. 2019), which is an important advantage for the production of aromatic compounds from highly recalcitrant biomass sources (Johnson et al. 2019; Lee et al. 2020). Nevertheless, the same metabolic pathways are not interesting when the bioprocess involve the conversion of glucose or glycerol into these molecules, requiring modifications in the Shikimate Pathway, which connects the central carbon metabolism pathways, where these carbon sources are incorporated, with the production of aromatic amino acids and derivatives with aromatic rings. Considering it in this study, in silico simulations based on the generation of elementary modes defined the best synthetic route to be introduced in the bacterial metabolism in order to produce GA from glucose or glycerol, through Shikimate Pathway intermediates. In this case, it is interesting to note that the elementary mode with the highest yield for glycerol consumption (Table 2) depended on the production of NADPH by transhydrogenases, instead of relying on the NADPH provided by the Pentose Phosphate Pathway (Table S3), one of the expected advantages of *P. putida*’s complex central carbon metabolism. It points out the fact that these enzymes might play an important role not only in the degradation of aromatic compounds by this species, as previously described (Nikel et al. 2016) but also in their production by novel strains, being interesting targets for future Metabolic Engineering manipulations. On the other hand, recent studies showed that modifications in *P. putida*’s central carbon can also be applied in order to favor the metabolic flux through the Shikimate Pathway, with the objective to produce new molecules derived from its intermediates, such as muconic acid (Bentley et al. 2020).

As described by Nikel et al. (2015b), the consumption of glycerol in *P. putida* KT2440 is regulated by the transcription factor GlpR which inhibits the expression of the operon *glp*FK and the gene *glp*D, both necessary for the transport and conversion of free glycerol into intermediates of the central metabolism of the cell. They showed that without the repressor, *P. putida* KT2440 Δ*glp*R became able to grow in a culture medium with glycerol as a sole carbon source at a faster rate than its parental strain. Because of that, the comparison of Gal1 with *E. coli* K12 MG1655 was important to highlight the potential of *P. putida* as a chassis for new bioprocesses involving the production of aromatic compounds from crude glycerol (Dias et al. 2017).

Regarding the results obtained from the production assays, many observations can be made. Firstly, when P_JAQP and Gal1_JAQP were compared (Fig. 3), it was interesting to note that despite both strains kept consuming glycerol over time, Gal1 was unable to grow after induction (24 h) as it used all the remaining glycerol to produce GA and PCA, while the parental strain was probably able to degrade these molecules and invest in biomass production or biopolymer accumulation (Beckers et al. 2016). This observation is important in terms of bioprocess evaluation, given that even with a final GA concentration of 346.7 ± 0.004 mg L^−1^, when the consumption of glycerol between the induction and the end of the assay (24–72 h) was taken into consideration, the yield obtained was 0.12 g/g (15,4% of the maximum yield predicted by the *in silico* analysis — Table 1), which is the same value observed by Kambourakis et al. (2000) in *E. coli* when glucose was provided in a 48 h bioreactor assay. This indicates that future assays in bioreactors can potentially improve the results seen in shaker flasks, given that important factors such as oxygenation, pH and temperature are better controlled. Furthermore, in terms of Synthetic Biology, the interruption of growth after induction also means that using vectors with robust systems whose promoters do not “leak” the expression in early stages of growth is crucial for the success of GA production and any other aromatic compounds coming from the Shikimate Pathway. In future developments, for example, if the use of inducers such as L-arabinose results in cost pressures to escalate the microbial production of GA in bioreactors, auto-inducible promoters that are cell-density sensitive could be tested (Meyers et al. 2019) or also promoters induced by nutrient limitation (Syn et al. 2004).

Secondly, when Gal1_JQP and Gal1_JAQP were compared (Fig. 5), the cells with plasmid JQP were able to produce more biomass than the control (Gal1_J0), consuming all glycerol available. This could be a result of a possible more favorable redox balance provided by the recovery of NADP in the synthetic route when the cell was still able to regulate its metabolic flux through the Shikimate Pathway and produce its aromatic amino acids. It is also important to point out the fact that Gal1 does not have the genes *gal*T and *gal*P, which encode a transporter and a porin, respectively (Nogales et al. 2011). This indicates that GA might be transported out of the cell by other means, probably by using the same transport mechanisms available for PCA transportation, as the protein sequences of both genes tend to align with orthologues related to PCA transportation in *P. putida* (pseudomonas.com). A recent study demonstrated that the 4-hydroxybenzoate transporter (PcaK) can also use 4HB as a substrate (Wada et al. 2021) so the possibility of GA being another substrate is something to be considered and investigated. At the same time, the fact that PCA was also released in similar or higher concentrations as GA highlights that the activity of the mutant enzyme PobA* might not be very efficient, being another possible target for improvements, as suggested by Moriwaki et al. (2019). However, if somehow not enough reductive power (NADPH) was generated *in vivo*, in contrary to the predictions made by the *in silico* model, this conversion would also not be favored, providing the same results.

Lastly, another important question to be addressed is the improvement of GA tolerance by *P. putida*. A recent study reported a certain level of GA toxicity to *P. putida* (Notonier et al. 2021) as it normally happens for many aromatic compounds. Due to that, techniques such as adaptive laboratory evolution (ALE) are already being employed for better production of high-value aromatic compounds, such as anthranilate, by *P. putida* and other species (Kuepper et al. 2020).

Based on all these results, it is possible to conclude that from mapping all reactions available in the metabolism of the cell of interest and gathering possible pathways that could be inserted synthetically by the expression of heterologous genes, *in silico* analyses were able to point out which genes should be selected for the production of GA by *P. putida*, thereby reducing the number of constructions to be tested and indicating future developments that might improve results through Metabolic Engineering. Then, combining the use of genome editing techniques and the construction of plasmids containing synthetic operons with site-directed mutations, the GA metabolism of *P. putida* KT2440, could be reversed, turning it from a consumer of GA into a producer. Thereby, by adopting GA as a model aromatic compound and making *Pseudomonas putida* KT2440 able to produce it from glycerol, the present study not only reinforced the potential of this strain as a SynBio chassis, but also built a proof of concept for future developments that aim the creation of new biorefineries capable to produce high value chemicals of medical interest in a sustainable way.

## Supplementary Information

Below is the link to the electronic supplementary material.

Supplementary file1 (DOCX 2.23 MB) **Supplementary information** Code lines used for the *in silico* analysis; Elementary modes obtained in the *in silico* analysis; Primers used; Examples of a chromatogram outcomes; Visual evidence of the production of GA by the new *P. putida *strain.

## Data Availability

Datasets, chromatograms, MetaTool output files and other data generated during this study are available from the corresponding author upon a request.

## Declarations

### Notes

This project resulted in a patent submission in Brazilian territory in 2020 (No. BR 10 2020 004349 8).
