# Correction to: From degrader to producer: reversing the gallic acid metabolism of *Pseudomonas putida* KT2440

**DOI:** 10.1007/s10123-023-00344-2

**Published:** 2023-03-02

**Authors:** Felipe M. S. Dias, Raoní K. Pantoja, José Gregório C. Gomez, Luiziana F. Silva

**Affiliations:** 1 Department of Microbiology, Institute of Biomedical Sciences, University of São Paulo, São Paulo, 05508-900 Brazil; 2 Centre for Genomic Regulation (CRG), The Barcelona Institute of Science and Technology, Dr. Aiguader 88, 08003 Barcelona, Spain; 3 Universitat Pompeu Fabra (UPF), Barcelona, Spain

**Correction to: International Microbiology**

10.1007/s10123-022-00282-5

The authors regret that the Table 2 and Supplementary Table S3 that appears in their original article is incorrect.

**Table 2:** List of elementary modes containing the highest yields (g/g) per carbon source and synthetic route chosen

Carbon source	Synthetic Route for GA production	Elementary modes	Maximum yield (g/g)
Glycerol	Route 1	54	0.7745
Glycerol	Routes 2 or 3	4, 5	0.6522
Glucose	Route 1	76, 98	0.7636
Glucose	Routes 2 or 3	73, 74, 81, 82, 91, 92, 93, 94	0.6408

Supplementary Table S3: List of elementary modes with the highest maximum yields and the fluxes in each reaction that compose them, according to the output file generated by Metatool. AD (1-2): Reactions that turn Pyruvate (PIR) into 2-Phosphoenolpyruvate (PEP); AS (1-7): Reactions from Shikimic Acid Pathway; CK (1-8): Reactions from the Citric Acid Cycle; ED (1-2): Reactions from the Entner-Doudoroff pathway; EMP (1-10): Reactions from the Embden-Meyerhof Parnas pathway; GA_SINT (1-6): Possible reactions included for gallic acid production using intermediates from the shikimic acid pathway. GA_SINT1 and 2 are the reactions that make Route 1, GA_SINT3, 5 and 2 make Route 2 and GA_SINT4, 6 and 2 make Route 3; GLN3: Single reaction that turns Fructose 1,6-bisphosphate (F16P) into F6P; GLY (1-2): Reactions from the acquisition and transformation of external Glycerol (Glyext) into Fructose-6-phosphate (F6P); OXFAD and OXNAD: Reactions that represent the oxidation of FADH and NADH, respectively; STHA_PNTAB: Reactions catalyzed by the transhydrogenases PntAB or SthA; VP (1-10): Reactions from the Pentose Phosphate Pathway:

**Table:** 

Carbon source	Synthetic Route for GA production	Elementary mode	Reactions and metabolic fluxes
Glycerol	Route 1	54	(31) (1.26667 GLN3) (0.2 EMP10) (9.06667 GLY) (3.8 AS1) (3.8 AS2) (3.8 GA_SINT1) (3.8 GA_SINT2) (0.2 CK1) (0.2 CK2) (0.2 CK3) (0.2 CK4) (0.2 CK5) (0.2 CK6) (0.2 CK7) (0.2 CK8) (0.2 CPD) OXNAD (0.2 OXFAD) (-1.26667 EMP4) (7.8 EMP5) (4 EMP6) (4 EMP7) (4 EMP8) (4 EMP9) (1.26667 VP6) (-1.26667 VP7) (1.26667 VP8) (1.26667 VP9) (-2.53333 VP10) (3.8 AS3) (-3.6 STHA_PNTAB) irreversible
Glycerol	Route 3	4	(37) (0.333333 GLN3) (2.83333 GLY) AS1 AS2 GA_SINT2 AS5 AS7 (0.5 CK1) (0.5 CK2) (0.5 CK3) (0.5 CK4) (0.5 CK5) (0.5 CK6) (0.5 CK7) (0.5 CK8) (0.5 CPD) (0.5 OXNAD) (0.5 OXFAD) (0.5 AD1) (0.5 AD2) (-0.333333 EMP4) (2.5 EMP5) (1.5 EMP6) (1.5 EMP7) (1.5 EMP8) (1.5 EMP9) (0.333333 VP6) (-0.333333 VP7) (0.333333 VP8) (0.333333 VP9) (-0.666667 VP10) AS3 AS4 AS6 GA_SINT4 GA_SINT6 (-2.5 STHA_PNTAB) irreversible
Glycerol	Route 2	5	(37) (0.266667 GLN3) (2.26667 GLY) (0.8 AS1) (0.8 AS2) (0.8 GA_SINT2) (0.8 AS5) (0.8 AS7) (0.4 CK1) (0.4 CK2) (0.4 CK3) (0.4 CK4) (0.4 CK5) (0.4 CK6) (0.4 CK7) (0.4 CK8) (0.4 CPD) (0.4 OXNAD) (0.4 OXFAD) (0.4 AD1) (0.4 AD2) (-0.266667 EMP4) (2 EMP5) (1.2 EMP6) (1.2 EMP7) (1.2 EMP8) (1.2 EMP9) (0.266667 VP6) (-0.266667 VP7) (0.266667 VP8) (0.266667 VP9) (-0.533333 VP10) (0.8 AS3) (0.8 AS4) (0.8 AS6) (0.8 GA_SINT3) (0.8 GA_SINT5) (-2 STHA_PNTAB) irreversible
Glucose	Route 1	76	(33) EMP1 (0.730496 ED1) (0.730496 ED2) (0.730496 VP1) (0.808511 AS1) (0.808511 AS2) (0.808511 GA_SINT1) (0.808511 GA_SINT2) (0.113475 CK1) (0.113475 CK2) (0.113475 CK3) (0.113475 CK4) (0.113475 CK5) (0.113475 CK6) (0.113475 CK7) (0.113475 CK8) (0.113475 CPD) (0.567376 OXNAD) (0.113475 OXFAD) (0.617021 AD1) (0.617021 AD2) (0.269504 EMP2) (0.191489 EMP6) (0.191489 EMP7) (0.191489 EMP8) (0.191489 EMP9) (0.269504 VP6) (-0.269504 VP7) (0.269504 VP8) (0.269504 VP9) (-0.539007 VP10) (0.808511 AS3) (0.035461 STHA_PNTAB) irreversible
Glucose	Route 1	98	(24) EMP1 (0.617021 ED1) (0.617021 ED2) (0.957447 VP1) (0.340426 VP5) (0.808511 AS1) (0.808511 AS2) (0.808511 GA_SINT1) (0.808511 GA_SINT2) (0.680851 OXNAD) (0.617021 AD1) (0.617021 AD2) (0.0425532 EMP2) (0.191489 EMP6) (0.191489 EMP7) (0.191489 EMP8) (0.191489 EMP9) (0.382979 VP6) (-0.0425532 VP7) (0.382979 VP8) (0.382979 VP9) (-0.425532 VP10) (0.808511 AS3) (0.489362 STHA_PNTAB) irreversible
Glucose	Route 3	73	(38) (1.47368 EMP1) (1.14035 ED1) (1.14035 ED2) (1.14035 VP1) AS1 AS2 GA_SINT2 AS5 AS7 (0.614035 CK1) (0.614035 CK2) (0.614035 CK3) (0.614035 CK4) (0.614035 CK5) (0.614035 CK6) (0.614035 CK7) (0.614035 CK8) (0.614035 CPD) (1.07018 OXNAD) (0.614035 OXFAD) (1.52632 AD1) (1.52632 AD2) (0.333333 EMP2) (0.473684 EMP6) (0.473684 EMP7) (0.473684 EMP8) (0.473684 EMP9) (0.333333 VP6) (-0.333333 VP7) (0.333333 VP8) (0.333333 VP9) (-0.666667 VP10) AS3 AS4 AS6 GA_SINT4 GA_SINT6 (-1.24561 STHA_PNTAB) irreversible
Glucose	Route 2	74	74: (38) (0.965517 EMP1) (0.747126 ED1) (0.747126 ED2) (0.747126 VP1) (0.655172 AS1) (0.655172 AS2) (0.655172 GA_SINT2) (0.655172 AS5) (0.655172 AS7) (0.402299 CK1) (0.402299 CK2) (0.402299 CK3) (0.402299 CK4) (0.402299 CK5) (0.402299 CK6) (0.402299 CK7) (0.402299 CK8) (0.402299 CPD) (0.701149 OXNAD) (0.402299 OXFAD) AD1 AD2 (0.218391 EMP2) (0.310345 EMP6) (0.310345 EMP7) (0.310345 EMP8) (0.310345 EMP9) (0.218391 VP6) (-0.218391 VP7) (0.218391 VP8) (0.218391 VP9) (-0.436782 VP10) (0.655172 AS3) (0.655172 AS4) (0.655172 AS6) (0.655172 GA_SINT3) (0.655172 GA_SINT5) (-0.816092 STHA_PNTAB) irreversible
Glucose	Route 3	81	(29) (1.47368 EMP1) (0.526316 ED1) (0.526316 ED2) (2.36842 VP1) (1.84211 VP5) AS1 AS2 GA_SINT2 AS5 AS7 (1.68421 OXNAD) (1.52632 AD1) (1.52632 AD2) (-0.894737 EMP2) (0.473684 EMP6) (0.473684 EMP7) (0.473684 EMP8) (0.473684 EMP9) (0.947368 VP6) (0.894737 VP7) (0.947368 VP8) (0.947368 VP9) (-0.0526316 VP10) AS3 AS4 AS6 GA_SINT4 GA_SINT6 (1.21053 STHA_PNTAB) irreversible
Glucose	Route 2	82	(29) (0.622222 EMP1) (0.222222 ED1) (0.222222 ED2) VP1 (0.777778 VP5) (0.422222 AS1) (0.422222 AS2) (0.422222 GA_SINT2) (0.422222 AS5) (0.422222 AS7) (0.711111 OXNAD) (0.644444 AD1) (0.644444 AD2) (-0.377778 EMP2) (0.2 EMP6) (0.2 EMP7) (0.2 EMP8) (0.2 EMP9) (0.4 VP6) (0.377778 VP7) (0.4 VP8) (0.4 VP9) (-0.0222222 VP10) (0.422222 AS3) (0.422222 AS4) (0.422222 AS6) (0.422222 GA_SINT3) (0.422222 GA_SINT5) (0.511111 STHA_PNTAB) irreversible
Glucose	Route 3	91	(37) (6.06811 EMP1) (4.00929 ED1) (4.00929 ED2) (6.06811 VP1) (2.05882 VP5) (4.11765 AS1) (4.11765 AS2) (4.11765 GA_SINT2) (4.11765 AS5) (4.11765 AS7) (1.84211 CK1) (1.84211 CK2) (1.84211 CK3) (1.84211 CK4) (1.84211 CK5) (1.84211 CK6) (1.84211 CK7) (1.84211 CK8) (1.84211 CPD) (5.09288 OXNAD) (1.84211 OXFAD) (6.28483 AD1) (6.28483 AD2) (1.95046 EMP6) (1.95046 EMP7) (1.95046 EMP8) (1.95046 EMP9) (2.05882 VP6) (2.05882 VP8) (2.05882 VP9) (-2.05882 VP10) (4.11765 AS3) (4.11765 AS4) (4.11765 AS6) (4.11765 GA_SINT4) (4.11765 GA_SINT6) (-2.3839 STHA_PNTAB) irreversible
Glucose	Route 2	92	(37) (6.06811 EMP1) (4.00929 ED1) (4.00929 ED2) (6.06811 VP1) (2.05882 VP5) (4.11765 AS1) (4.11765 AS2) (4.11765 GA_SINT2) (4.11765 AS5) (4.11765 AS7) (1.84211 CK1) (1.84211 CK2) (1.84211 CK3) (1.84211 CK4) (1.84211 CK5) (1.84211 CK6) (1.84211 CK7) (1.84211 CK8) (1.84211 CPD) (5.09288 OXNAD) (1.84211 OXFAD) (6.28483 AD1) (6.28483 AD2) (1.95046 EMP6) (1.95046 EMP7) (1.95046 EMP8) (1.95046 EMP9) (2.05882 VP6) (2.05882 VP8) (2.05882 VP9) (-2.05882 VP10) (4.11765 AS3) (4.11765 AS4) (4.11765 AS6) (4.11765 GA_SINT3) (4.11765 GA_SINT5) (-2.3839 STHA_PNTAB) irreversible
Glucose	Route 3	93	(38) (3.76285 EMP1) (2.1166 ED1) (2.1166 ED2) (4.50198 VP1) (2.38538 VP5) (2.55336 AS1) (2.55336 AS2) (2.55336 GA_SINT2) (2.55336 AS5) (2.55336 AS7) (0.772727 CK1) (0.772727 CK2) (0.772727 CK3) (0.772727 CK4) (0.772727 CK5) (0.772727 CK6) (0.772727 CK7) (0.772727 CK8) (0.772727 CPD) (3.52767 OXNAD) (0.772727 OXFAD) (3.89723 AD1) (3.89723 AD2) (-0.73913 EMP2) (1.20949 EMP6) (1.20949 EMP7) (1.20949 EMP8) (1.20949 EMP9) (1.64625 VP6) (0.73913 VP7) (1.64625 VP8) (1.64625 VP9) (-0.907115 VP10) (2.55336 AS3) (2.55336 AS4) (2.55336 AS6) (2.55336 GA_SINT4) (2.55336 GA_SINT6) irreversible
Glucose	Route 2	94	(38) (2.40049 EMP1) (1.35028 ED1) (1.35028 ED2) (2.87201 VP1) (1.52174 VP5) (1.6289 AS1) (1.6289 AS2) (1.6289 GA_SINT2) (1.6289 AS5) (1.6289 AS7) (0.492958 CK1) (0.492958 CK2) (0.492958 CK3) (0.492958 CK4) (0.492958 CK5) (0.492958 CK6) (0.492958 CK7) (0.492958 CK8) (0.492958 CPD) (2.25046 OXNAD) (0.492958 OXFAD) (2.48622 AD1) (2.48622 AD2) (-0.471525 EMP2) (0.771586 EMP6) (0.771586 EMP7) (0.771586 EMP8) (0.771586 EMP9) (1.05021 VP6) (0.471525 VP7) (1.05021 VP8) (1.05021 VP9) (-0.57869 VP10) (1.6289 AS3) (1.6289 AS4) (1.6289 AS6) (1.6289 GA_SINT3) (1.6289 GA_SINT5) irreversible

The original article has been corrected.

