# Supplementary material for: From degrader to producer: reversing the gallic acid metabolism of Pseudomonas putida KT2440
Source: Int Microbiol. 2022 Nov 11;26(2):243–55. doi: 10.1007/s10123-022-00282-5 (PMC9649394; doi:10.1007/s10123-022-00282-5)
Supplement: Supplementary file 1 — Supplementary file1 (DOCX 2.23 MB) Supplementary information Code lines used for the in silico analysis; Elementary modes obtained in the in silico analysis; Primers used; Examples of a chromatogram outcomes; Visual evidence of the production of GA by the new P. putida strain. [file 10123_2022_282_MOESM1_ESM.docx]

International Microbiology

From degrader to producer: Reversing the gallic acid metabolism of *Pseudomonas putida* KT2440

Felipe M. S. Dias*^1^, Raoní K. Pantoja^1^, José Gregório C. Gomez^1^, Luiziana F. Silva^1^

^1^Department of Microbiology, Institute of Biomedical Sciences, University of São Paulo, 05508-888, São Paulo, Brazil

*E-mail address: felipe.morillo@crg.eu, telephone number: +34 645 48 26 06

[Supplementary Tool S1: Input file for Metatool: Glycerol as the sole carbon source 3](#_heading=h.gjdgxs)

[Supplementary Tool S2: Input file for Metatool: Glucose as the sole carbon source](#_heading=h.30j0zll) 5

Supplementary Table S1: Elementary modes obtained: Glycerol as the sole carbon source 8

Supplementary Table S2: Elementary modes obtained: Glucose as the sole carbon source 12

Supplementary Table S3: List of elementary modes with maximum yields26

Supplementary Table S4: List of primers used29

Supplementary Figure S1. Electrophoresis gels to validate insertions 31

Supplementary Figure S2. Electrophoresis gels to validate deletions32

Supplementary Figure S3: Chromatograms for gallic and protocatechuic acids (2^nd^ assay)33

Supplementary Figure S4: Chromatograms for gallic and protocatechuic acids (3^rd^ assay)34

Supplementary Figure S5: Oxidation of the culture medium due to gallic acid 34

**Supplementary Tool S1**: Code lines used in the input file for the MetaTool in silico analysis, considering only glycerol as a carbon source. ENZREV and ENZIRRREV: All reversible and irreversible chemical reactions, respectively; AD (1-2): Reactions that turn Pyruvate (PIR) into 2-Phosphoenolpyruvate (PEP); AS (1-7): Reactions from Shikimic Acid Pathway; CK (1-8): Reactions from the Citric Acid Cycle; ED (1-2): Reactions from the Entner-Doudoroff pathway; EMP (2-10): Reactions from the Embden-Meyerhof Parnas pathway; GA_SINT (1-6): Possible reactions included for gallic acid production using intermediates from the shikimic acid pathway. GA_SINT1 and 2 are the reactions that make Route 1, GA_SINT3, 5 and 2 make Route 2 and GA_SINT4, 6 and 2 make Route 3; GLN3: Single reaction that turns Fructose 1,6-bisphosphate (F16P) into F6P; GLY: Reaction from the acquisition and transformation of external Glycerol (Glyext) into Dihydroxyacetone-Phosphate (DHP); OXFAD and OXNAD: Reactions that represent the oxidation of FADH and NADH, respectively; STHA_PNTAB: Reactions catalyzed by the transhydrogenases PntAB or SthA; VP (1-10): Reactions from the Pentose Phosphate Pathway; METINT and METEXT: All internal and external metabolites and other molecules, respectively; 3HB: 3-Hydroxybenzoate; 4HB: 4-Hydroxybenzoate; AcCoa: Acetyl-Coenzyme A; aCeto: 2-Ketoglutarate; ADP: Adenosine diphosphate; ATP: Adenosine triphosphate; BPG13: Glycerate-1,3-Biphosphate; CHO: Chorismate; Cit: Citrate; CO2: Carbon Dioxide; CoASH: Reduced form of Coenzyme A; DHAP: 3-Deoxy-D-arabino-heptulosonate 7-phosfate; DHP: Dihydroxyacetone-Phosphate; DHQ: 3-Dehydroquinate; DHS: 3-Dehydroshikimate; E4P: Eryhtrose-4-Phosphate; EPSP: 5-enolpyruvylshikimate-3-phosphate; F16P: Fructose-1,6-bisphosphate; F6P: Fructose-6-Phosphate; FAD: Flavin adenine dinucleotide; FADH2: Hydroquinone form of FAD; Fuma: Fumarate; G3P: Glyceraldehyde-3-Phosphate; G6P: Glucose 6-phosphate; GA: Gallic acid; Glyext: Glycerol; IsoCit: Isocitrate; KDPG2: 2-keto-3-deoxy-6-phosphogluconate; Mal: Malate; NAD: Nicotinamide adenine dinucleotide; NADH: Reduced form of NAD; NADP: Nicotinamide adenine dinucleotide phosphate; NADPH: Reduced form of NADP; Oxa: Oxalacetate; O: Oxygen; PCA: Protocatechuate; PEP: Phosphoenolpyruvate; PG2: Glycerate-2-Phosphate; PG3: DGlycerate-3-Phosphate; PG6: 6-Phosphogluconate; PIR: Pyruvate; Rb5P: Ribose-5-Phosphate; Rbl5P: Ribulose-5-Phosphate; X5P: Xylulose-5-phosphate; S3P: Shikimate-3-Phosphate; S7P: Sedoheptulose-7-Phosphate; SHI: Shikimate; Suc: Succinate; SucCoa: Succinyl-Coenzyme A.

# GA Production in P putida KT2440 (GLYCEROL)

-ENZREV

EMP2 EMP4 EMP5 EMP6 EMP7 EMP8 EMP9 VP6 VP7 VP8 VP9 VP10 AS3 AS4 AS6 GA_SINT3 GA_SINT4 GA_SINT5 GA_SINT6 STHA_PNTAB

-ENZIRREV

GLN3 EMP1 EMP10 ED1 ED2 VP1 VP5 GLY AS1 AS2 GA_SINT1 GA_SINT2 AS5 AS7 CK1 CK2 CK3 CK4 CK5 CK6 CK7 CK8 CPD OXNAD OXFAD AD1 AD2

-METINT

G6P PG6 KDPG2 Rbl5P Rb5P X5P S7P E4P F6P F16P DHP G3P BPG13 PG3 PG2 PEP DHAP DHQ DHS PCA SHI S3P EPSP CHO 4HB 3HB NADP NADPH NAD NADH PIR CoASH AcCoa Oxa Cit IsoCit aCeto SucCoa Suc Fuma Mal FAD FADH2 ADP ATP

-METEXT

Glyext CO2 O GA

-CAT

GLY : Glyext + ATP = DHP + ADP .

GLN3 : F16P + ADP = F6P + ATP .

EMP2 : G6P = F6P .

EMP4 : F16P = G3P + DHP .

EMP5 : DHP = G3P .

EMP6 : G3P + NAD = BPG13 + NADH .

EMP7 : BPG13 + ADP = PG3 + ATP .

EMP8 : PG3 = PG2 .

EMP9 : PG2 = PEP .

EMP10 : PEP + ADP = PIR + ATP .

ED1 : PG6 = KDPG2 .

ED2 : KDPG2 = PIR + G3P .

VP1 : G6P + NADP = PG6 + NADPH .

VP5 : PG6 + NADP = NADPH + Rbl5P + CO2 .

VP6 : Rbl5P = Rb5P .

VP7 : Rbl5P = X5P .

VP8 : Rb5P + X5P = S7P + G3P .

VP9 : G3P + S7P = E4P + F6P .

VP10 : X5P + E4P = F6P + G3P .

AS1 : E4P + PEP = DHAP .

AS2 : DHAP = DHQ .

AS3 : DHQ = DHS .

GA_SINT1 : DHS = PCA .

GA_SINT2 : PCA + NADPH + O = GA + NADP .

AS4 : DHS + NADPH = SHI + NADP .

AS5 : SHI + ATP = S3P + ADP .

AS6 : S3P + PEP = EPSP .

AS7 : EPSP = CHO .

GA_SINT3 : CHO = PIR + 4HB .

GA_SINT4 : CHO = PIR + 3HB .

GA_SINT5 : 4HB + O + NADPH = PCA + NADP .

GA_SINT6 : 3HB + O + NADPH = PCA + NADP .

CPD : PIR + NAD + CoASH = AcCoa + NADH + CO2 .

CK1 : AcCoa + Oxa = CoASH + Cit .

CK2 : Cit = IsoCit .

CK3 : IsoCit + NADP = aCeto + NADPH + CO2 .

CK4 : CoASH + aCeto + NAD = NADH + CO2 + SucCoa .

CK5 : SucCoa + ADP = CoASH + Suc + ATP .

CK6 : Suc + FAD = FADH2 + Fuma .

CK7 : Fuma = Mal .

CK8 : Mal + NAD = Oxa + NADH .

OXNAD : NADH + 3 ADP + O = NAD + 3 ATP .

OXFAD : FADH2 + 2 ADP + O = FAD + 2 ATP .

STHA_PNTAB : NADPH + NAD = NADP + NADH .

AD1 : PIR + CO2 + ATP = Oxa + ADP .

AD2 : Oxa + ATP = PEP + ADP + CO2 .

**Supplementary Tool S2**: Code lines used in the input file for the MetaTool in silico analysis, considering only glucose as a carbon source. ENZREV and ENZIRRREV: All reversible and irreversible chemical reactions, respectively; AD (1-2): Reactions that turn Pyruvate (PIR) into 2-Phosphoenolpyruvate (PEP); AS (1-7): Reactions from Shikimic Acid Pathway; CK (1-8): Reactions from the Citric Acid Cycle; ED (1-2): Reactions from the Entner-Doudoroff pathway; EMP (1-10): Reactions from the Embden-Meyerhof Parnas pathway; GA_SINT (1-6): Possible reactions included for gallic acid production using intermediates from the shikimic acid pathway. GA_SINT1 and 2 are the reactions that make Route 1, GA_SINT3, 5 and 2 make Route 2 and GA_SINT4, 6 and 2 make Route 3; GLN3: Single reaction that turns Fructose 1,6-bisphosphate (F16P) into F6P; OXFAD and OXNAD: Reactions that represent the oxidation of FADH and NADH, respectively; PR (1-5): Reactions that represent peripheral catalysis of Glucose (Gluext) into 6-Phosphogluconate (PG6); STHA_PNTAB: Reactions catalyzed by the transhydrogenases PntAB or SthA; VP (1-10): Reactions from the Pentose Phosphate Pathway; METINT and METEXT: All internal and external metabolites and other molecules, respectively; 3HB: 3-Hydroxybenzoate; 4HB: 4-Hydroxybenzoate; AcCoa: Acetyl-Coenzyme A; aCeto: 2-Ketoglutarate; ADP: Adenosine diphosphate; ATP: Adenosine triphosphate; BPG13: Glycerate-1,3-Biphosphate; CHO: Chorismate; Cit: Citrate; CO2: Carbon Dioxide; CoASH: Reduced form of Coenzyme A; DHAP: 3-Deoxy-D-arabino-heptulosonate 7-phosfate; DHP: Dihydroxyacetone-Phosphate; DHQ: 3-Dehydroquinate; DHS: 3-Dehydroshikimate; E4P: Eryhtrose-4-Phosphate; EPSP: 5-enolpyruvylshikimate-3-phosphate; F16P: Fructose-1,6-bisphosphate; F6P: Fructose-6-Phosphate; FAD: Flavin adenine dinucleotide; FADH2: Hydroquinone form of FAD; Fuma: Fumarate; G3P: Glyceraldehyde-3-Phosphate; G6P: Glucose 6-phosphate; GA: Gallic acid; GC: Gluconate; Gluext: Glucose; IsoCit: Isocitrate; KDPG2: 2-keto-3-deoxy-6-phosphogluconate; Mal: Malate; NAD: Nicotinamide adenine dinucleotide; NADH: Reduced form of NAD; NADP: Nicotinamide adenine dinucleotide phosphate; NADPH: Reduced form of NADP; Oxa: Oxalacetate; O: Oxygen; PCA: Protocatechuate; PEP: Phosphoenolpyruvate; PG2: Glycerate-2-Phosphate; PG3: DGlycerate-3-Phosphate; PG6: 6-Phosphogluconate; PIR: Pyruvate; K6PG: 2-ketogluconate-6-phosphate; KGC: 2-ketogluconate; Rb5P: Ribose-5-Phosphate; Rbl5P: Ribulose-5-Phosphate; X5P: Xylulose-5-phosphate; S3P: Shikimate-3-Phosphate; S7P: Sedoheptulose-7-Phosphate; SHI: Shikimate; Suc: Succinate; SucCoa: Succinyl-Coenzyme A.

# GA Production in P putida KT2440 (GLUCOSE)

#

#

#

#

-ENZREV

EMP2 EMP4 EMP5 EMP6 EMP7 EMP8 EMP9 VP6 VP7 VP8 VP9 VP10 AS3 AS4 AS6 GA_SINT3 GA_SINT4 GA_SINT5 GA_SINT6 STHA_PNTAB

-ENZIRREV

GLN3 EMP1 EMP10 ED1 ED2 VP1 VP5 AS1 AS2 GA_SINT1 GA_SINT2 AS5 AS7 CK1 CK2 CK3 CK4 CK5 CK6 CK7 CK8 CPD OXNAD OXFAD AD1 AD2 PR1 PR2 PR3 PR4 PR5

-METINT

GC KGC K6PG G6P PG6 KDPG2 Rbl5P Rb5P X5P S7P E4P F6P F16P DHP G3P BPG13 PG3 PG2 PEP DHAP DHQ DHS PCA SHI S3P EPSP CHO 4HB 3HB NADP NADPH NAD NADH PIR CoASH AcCoa Oxa Cit IsoCit aCeto SucCoa Suc Fuma Mal FAD FADH2 ADP ATP

-METEXT

Gluext CO2 O GA

-CAT

PR1 : Gluext = GC .

PR2 : GC + FAD = KGC + FADH2 .

PR3 : KGC + ATP = K6PG + ADP .

PR4 : K6PG + NADPH = PG6 + NADP .

PR5 : GC + ATP = PG6 + ADP .

GLN3 : F16P + ADP = F6P + ATP .

EMP1 : Gluext + ATP = G6P + ADP .

EMP2 : G6P = F6P .

EMP4 : F16P = G3P + DHP .

EMP5 : DHP = G3P .

EMP6 : G3P + NAD = BPG13 + NADH .

EMP7 : BPG13 + ADP = PG3 + ATP .

EMP8 : PG3 = PG2 .

EMP9 : PG2 = PEP .

EMP10 : PEP + ADP = PIR + ATP .

ED1 : PG6 = KDPG2 .

ED2 : KDPG2 = PIR + G3P .

VP1 : G6P + NADP = PG6 + NADPH .

VP5 : PG6 + NADP = NADPH + Rbl5P + CO2 .

VP6 : Rbl5P = Rb5P .

VP7 : Rbl5P = X5P .

VP8 : Rb5P + X5P = S7P + G3P .

VP9 : G3P + S7P = E4P + F6P .

VP10 : X5P + E4P = F6P + G3P .

AS1 : E4P + PEP = DHAP .

AS2 : DHAP = DHQ .

AS3 : DHQ = DHS .

GA_SINT1 : DHS = PCA .

GA_SINT2 : PCA + NADPH + O = GA + NADP .

AS4 : DHS + NADPH = SHI + NADP .

AS5 : SHI + ATP = S3P + ADP .

AS6 : S3P + PEP = EPSP .

AS7 : EPSP = CHO .

GA_SINT3 : CHO = PIR + 4HB .

GA_SINT4 : CHO = PIR + 3HB .

GA_SINT5 : 4HB + O + NADPH = PCA + NADP .

GA_SINT6 : 3HB + O + NADPH = PCA + NADP .

CPD : PIR + NAD + CoASH = AcCoa + NADH + CO2 .

CK1 : AcCoa + Oxa = CoASH + Cit .

CK2 : Cit = IsoCit .

CK3 : IsoCit + NADP = aCeto + NADPH + CO2 .

CK4 : CoASH + aCeto + NAD = NADH + CO2 + SucCoa .

CK5 : SucCoa + ADP = CoASH + Suc + ATP .

CK6 : Suc + FAD = FADH2 + Fuma .

CK7 : Fuma = Mal .

CK8 : Mal + NAD = Oxa + NADH .

OXNAD : NADH + 3 ADP + O = NAD + 3 ATP .

OXFAD : FADH2 + 2 ADP + O = FAD + 2 ATP .

STHA_PNTAB : NADPH + NAD = NADP + NADH .

AD1 : PIR + CO2 + ATP = Oxa + ADP .

AD2 : Oxa + ATP = PEP + ADP + CO2 .

**Supplementary Table S1:** Elementary modes obtained from the *in silico* analysis, considering only glycerol as a carbon source and their overall reactions, according to the output file generated by Metatool. CO2: Carbon dioxide; GA: Gallic acid; Glyext: Glycerol; O: Oxigen (O_2_):

| **Elementary mode number** | **Overall reaction** |
| --- | --- |
| 1: | no net transformation of external metabolites |
| 2: | Glyext + 6 O = 3 CO2 |
| 3: | no net transformation of external metabolites |
| 4: | 2.83333 Glyext + 3 O = 1.5 CO2 + GA |
| 5: | 2.83333 Glyext + 3 O = 1.5 CO2 + GA |
| 6: | 8.88889 Glyext + 5.33333 O = CO2 + 3.66667 GA |
| 7: | 8.88889 Glyext + 5.33333 O = CO2 + 3.66667 GA |
| 8: | 2.88889 Glyext + 3.33333 O = 1.66667 CO2 + GA |
| 9: | 2.88889 Glyext + 3.33333 O = 1.66667 CO2 + GA |
| 10: | 2.85 Glyext + 3.1 O = 1.55 CO2 + GA |
| 11: | 2.85 Glyext + 3.1 O = 1.55 CO2 + GA |
| 12: | 2.88235 Glyext + 3.29412 O = 1.64706 CO2 + GA |
| 13: | 2.88235 Glyext + 3.29412 O = 1.64706 CO2 + GA |
| 14: | 14.3333 Glyext + 8 O = CO2 + 6 GA |
| 15: | 5 Glyext + 3.55556 O = CO2 + 2 GA |
| 16: | 5 Glyext + 3.55556 O = CO2 + 2 GA |
| 17: | 2.72222 Glyext + 2.66667 O = 1.16667 CO2 + GA |
| 18: | 2.72222 Glyext + 2.66667 O = 1.16667 CO2 + GA |
| 19: | Glyext + 6 O = 3 CO2 |
| 20: | 3.11111 Glyext + 4.66667 O = 2.33333 CO2 + GA |
| 21: | 3.11111 Glyext + 4.66667 O = 2.33333 CO2 + GA |
| 22: | 3 Glyext + 4 O = 2 CO2 + GA |
| 23: | 3 Glyext + 4 O = 2 CO2 + GA |
| 24: | 3.06061 Glyext + 4.36364 O = 2.18182 CO2 + GA |
| 25: | 3.06061 Glyext + 4.36364 O = 2.18182 CO2 + GA |
| 26: | 14.3333 Glyext + 8 O = CO2 + 6 GA |
| 27: | 3.13333 Glyext + 3.2 O = CO2 + 1.2 GA |
| 28: | 5 Glyext + 4 O = CO2 + 2 GA |
| 29: | 6.03704 Glyext + 4.44444 O = CO2 + 2.44444 GA |
| 30: | 2.66667 Glyext + 2.66667 O = CO2 + 1 GA |
| 31: | 2.66667 Glyext + 2.66667 O = CO2 + 1 GA |
| 32: | Glyext + 6 O = 3 CO2 |
| 33: | 3.11111 Glyext + 4.66667 O = 2.33333 CO2 + GA |
| 34: | 3.11111 Glyext + 4.66667 O = 2.33333 CO2 + GA |
| 35: | 3 Glyext + 4 O = 2 CO2 + GA |
| 36: | 3 Glyext + 4 O = 2 CO2 + GA |
| 37: | 3.11111 Glyext + 4.66667 O = 2.33333 CO2 + GA |
| 38: | 3.11111 Glyext + 4.66667 O = 2.33333 CO2 + GA |
| 39: | 3.11111 Glyext + 4.66667 O = 2.33333 CO2 + GA |
| 40: | 3.11111 Glyext + 4.66667 O = 2.33333 CO2 + GA |
| 41: | 3 Glyext + 4 O = 2 CO2 + GA |
| 42: | 3 Glyext + 4 O = 2 CO2 + GA |
| 43: | 3.13333 Glyext + 3.2 O = CO2 + 1.2 GA |
| 44: | 5 Glyext + 4 O = CO2 + 2 GA |
| 45: | 3 Glyext + 5 O = 2 CO2 + GA |
| 46: | 8.11111 Glyext + 5.33333 O = CO2 + 3.33333 GA |
| 47: | 3.13333 Glyext + 3.2 O = CO2 + 1.2 GA |
| 48: | 3 Glyext + 4.22222 O = 2 CO2 + GA |
| 49: | 3 Glyext + 4.22222 O = 2 CO2 + GA |
| 50: | 5 Glyext + 3.69231 O = CO2 + 2 GA |
| 51: | 5 Glyext + 3.69231 O = CO2 + 2 GA |
| 52: | 5.33333 Glyext + 18 O = 9 CO2 + GA |
| 53: | 5.33333 Glyext + 18 O = 9 CO2 + GA |
| 54: | 15.1111 Glyext + 8.33333 O = CO2 + 6.33333 GA |
| 55: | 3.33333 Glyext + 7 O = 3 CO2 + GA |
| 56: | Glyext + 6 O = 3 CO2 |
| 57: | 3 Glyext + 4 O = 2 CO2 + GA |
| 58: | 3 Glyext + 4 O = 2 CO2 + GA |
| 59: | 4.5 Glyext + 13 O = 6.5 CO2 + GA |
| 60: | 4.5 Glyext + 13 O = 6.5 CO2 + GA |
| 61: | 5 Glyext + 4 O = CO2 + 2 GA |
| 62: | 3 Glyext + 5 O = 2 CO2 + GA |
| 63: | Glyext + 6 O = 3 CO2 |
| 64: | 3.33333 Glyext + 6 O = 3 CO2 + GA |
| 65: | 3.33333 Glyext + 6 O = 3 CO2 + GA |
| 66: | 3 Glyext + 5 O = 2 CO2 + GA |
| 67: | 3 Glyext + 5 O = 2 CO2 + GA |

**Supplementary Table S2**: Elementary modes obtained from the in silico analysis, considering only glucose as a carbon source and their overall reactions, according to the output file generated by Metatool. CO2: Carbon dioxide; GA: Gallic acid; Gluext: Glucose; O: Oxigen (O_2_):

| **Elementary mode number** | **Overall reaction** |
| --- | --- |
| 1: | no net transformation of external metabolites |
| 2: | 1.63462 Gluext + 3.98077 O = 2.80769 CO2 + GA |
| 3: | 1.63462 Gluext + 3.98077 O = 2.80769 CO2 + GA |
| 4: | 1.6 Gluext + 3.6 O = 2.6 CO2 + GA |
| 5: | 1.6 Gluext + 3.6 O = 2.6 CO2 + GA |
| 6: | 1.36538 Gluext + 2.01923 O = 1.19231 CO2 + GA |
| 7: | 1.34286 Gluext + 1.77143 O = 1.05714 CO2 + GA |
| 8: | 1.6 Gluext + 3.6 O = 2.6 CO2 + GA |
| 9: | 1.6 Gluext + 3.6 O = 2.6 CO2 + GA |
| 10: | 1.64706 Gluext + 4.11765 O = 2.88235 CO2 + GA |
| 11: | 1.64706 Gluext + 4.11765 O = 2.88235 CO2 + GA |
| 12: | 1.64 Gluext + 4.04 O = 2.84 CO2 + GA |
| 13: | 1.64 Gluext + 4.04 O = 2.84 CO2 + GA |
| 14: | 1.64706 Gluext + 4.11765 O = 2.88235 CO2 + GA |
| 15: | 1.64706 Gluext + 4.11765 O = 2.88235 CO2 + GA |
| 16: | 1.6 Gluext + 3.6 O = 2.6 CO2 + GA |
| 17: | 1.6 Gluext + 3.6 O = 2.6 CO2 + GA |
| 18: | 1.6 Gluext + 3.6 O = 2.6 CO2 + GA |
| 19: | 1.6 Gluext + 3.6 O = 2.6 CO2 + GA |
| 20: | 1.64706 Gluext + 4.11765 O = 2.88235 CO2 + GA |
| 21: | 1.64706 Gluext + 4.11765 O = 2.88235 CO2 + GA |
| 22: | 1.60606 Gluext + 3.66667 O = 2.63636 CO2 + GA |
| 23: | 1.60606 Gluext + 3.66667 O = 2.63636 CO2 + GA |
| 24: | 1.375 Gluext + 2.125 O = 1.25 CO2 + GA |
| 25: | 1.34286 Gluext + 1.77143 O = 1.05714 CO2 + GA |
| 26: | 1.36 Gluext + 1.96 O = 1.16 CO2 + GA |
| 27: | 1.34286 Gluext + 1.77143 O = 1.05714 CO2 + GA |
| 28: | 1.45455 Gluext + 2.72727 O = 1.72727 CO2 + GA |
| 29: | 1.45455 Gluext + 2.72727 O = 1.72727 CO2 + GA |
| 30: | 1.5 Gluext + 2.88889 O = 2 CO2 + GA |
| 31: | 1.5 Gluext + 2.88889 O = 2 CO2 + GA |
| 32: | 1.5 Gluext + 3.05556 O = 2 CO2 + GA |
| 33: | 1.5 Gluext + 3.05556 O = 2 CO2 + GA |
| 34: | 1.5 Gluext + 3 O = 2 CO2 + GA |
| 35: | 1.5 Gluext + 3 O = 2 CO2 + GA |
| 36: | Gluext + 11 O = 6 CO2 |
| 37: | Gluext + 11 O = 6 CO2 |
| 38: | 2 Gluext + 8 O = 5 CO2 + GA |
| 39: | 2 Gluext + 8 O = 5 CO2 + GA |
| 40: | 2 Gluext + 8 O = 5 CO2 + GA |
| 41: | 2 Gluext + 8 O = 5 CO2 + GA |
| 42: | 2 Gluext + 9 O = 5 CO2 + GA |
| 43: | 2 Gluext + 9 O = 5 CO2 + GA |
| 44: | Gluext + 11 O = 6 CO2 |
| 45: | Gluext + 11 O = 6 CO2 |
| 46: | 1.6875 Gluext + 4.5625 O = 3.125 CO2 + GA |
| 47: | 1.6875 Gluext + 4.5625 O = 3.125 CO2 + GA |
| 48: | 2.5 Gluext + 13.5 O = 8 CO2 + GA |
| 49: | 2.5 Gluext + 13.5 O = 8 CO2 + GA |
| 50: | 5 Gluext + 41 O = 23 CO2 + GA |
| 51: | 5 Gluext + 41 O = 23 CO2 + GA |
| 52: | 1.4375 Gluext + 2.8125 O = 1.625 CO2 + GA |
| 53: | 1.5 Gluext + 3.5 O = 2 CO2 + GA |
| 54: | 3 Gluext + 20 O = 11 CO2 + GA |
| 55: | 1.66667 Gluext + 4.33333 O = 3 CO2 + GA |
| 56: | 1.66667 Gluext + 4.33333 O = 3 CO2 + GA |
| 57: | 3 Gluext + 19 O = 11 CO2 + GA |
| 58: | 3 Gluext + 19 O = 11 CO2 + GA |
| 59: | 1.4 Gluext + 2.4 O = 1.4 CO2 + GA |
| 60: | 1.5 Gluext + 3.5 O = 2 CO2 + GA |
| 61: | 1.5 Gluext + 3.5 O = 2 CO2 + GA |
| 62: | 1.44444 Gluext + 2.88889 O = 1.66667 CO2 + GA |
| 63: | 1.5 Gluext + 3.25 O = 2 CO2 + GA |
| 64: | 1.5 Gluext + 3.25 O = 2 CO2 + GA |
| 65: | 1.60256 Gluext + 3.96154 O = 2.61538 CO2 + GA |
| 66: | 1.60256 Gluext + 3.96154 O = 2.61538 CO2 + GA |
| 67: | 1.57143 Gluext + 3.61905 O = 2.42857 CO2 + GA |
| 68: | 1.57143 Gluext + 3.61905 O = 2.42857 CO2 + GA |
| 69: | 1.33333 Gluext + 2 O = CO2 + 1 GA |
| 70: | 1.48387 Gluext + 2.02151 O = CO2 + 1.12903 GA |
| 71: | 1.59259 Gluext + 3.98148 O = 2.55556 CO2 + GA |
| 72: | 1.59259 Gluext + 3.98148 O = 2.55556 CO2 + GA |
| 73: | 1.47368 Gluext + 3.68421 O = 1.84211 CO2 + GA |
| 74: | 1.47368 Gluext + 3.68421 O = 1.84211 CO2 + GA |
| 75: | 1.47917 Gluext + 2.29167 O = CO2 + 1.125 GA |
| 76: | 2.9375 Gluext + 4.375 O = CO2 + 2.375 GA |
| 77: | 2.76923 Gluext + 4.10256 O = CO2 + 2.23077 GA |
| 78: | 2.83333 Gluext + 4.22619 O = CO2 + 2.28571 GA |
| 79: | 2.65217 Gluext + 4.05797 O = CO2 + 2.13043 GA |
| 80: | 2.65217 Gluext + 4.05797 O = CO2 + 2.13043 GA |
| 81: | 1.47368 Gluext + 3.68421 O = 1.84211 CO2 + GA |
| 82: | 1.47368 Gluext + 3.68421 O = 1.84211 CO2 + GA |
| 83: | 1.5 Gluext + 3.66667 O = 2 CO2 + GA |
| 84: | 1.5 Gluext + 3.66667 O = 2 CO2 + GA |
| 85: | 1.5 Gluext + 3.75 O = 2 CO2 + GA |
| 86: | 1.5 Gluext + 3.75 O = 2 CO2 + GA |
| 87: | 1.625 Gluext + 4.0625 O = 2.75 CO2 + GA |
| 88: | 1.625 Gluext + 4.0625 O = 2.75 CO2 + GA |
| 89: | 1.61111 Gluext + 4.02778 O = 2.66667 CO2 + GA |
| 90: | 1.61111 Gluext + 4.02778 O = 2.66667 CO2 + GA |
| 91: | 1.47368 Gluext + 3.68421 O = 1.84211 CO2 + GA |
| 92: | 1.47368 Gluext + 3.68421 O = 1.84211 CO2 + GA |
| 93: | 1.47368 Gluext + 3.68421 O = 1.84211 CO2 + GA |
| 94: | 1.47368 Gluext + 3.68421 O = 1.84211 CO2 + GA |
| 95: | 1.5625 Gluext + 3.90625 O = 2.375 CO2 + GA |
| 96: | 1.5625 Gluext + 3.90625 O = 2.375 CO2 + GA |
| 97: | 1.35714 Gluext + 2.14286 O = 1.14286 CO2 + GA |
| 98: | 2.9375 Gluext + 4.375 O = CO2 + 2.375 GA |
| 99: | 2.5 Gluext + 3.66667 O = CO2 + 2 GA |
| 100: | 2.5 Gluext + 3.75 O = CO2 + 2 GA |
| 101: | 1.5 Gluext + 2.32143 O = CO2 + 1.14286 GA |
| 102: | 1.33333 Gluext + 2.08333 O = CO2 + 1 GA |
| 103: | 1.52 Gluext + 2.08 O = CO2 + 1.16 GA |
| 104: | 1.86364 Gluext + 2.84091 O = CO2 + 1.45455 GA |
| 105: | 2.5 Gluext + 3.88889 O = CO2 + 2 GA |
| 106: | 2.5 Gluext + 3.88889 O = CO2 + 2 GA |
| 107: | 1.5 Gluext + 3.25 O = 2 CO2 + GA |
| 108: | 1.5 Gluext + 3.25 O = 2 CO2 + GA |
| 109: | 1.36735 Gluext + 2.85714 O = 1.20408 CO2 + GA |
| 110: | 1.36735 Gluext + 2.85714 O = 1.20408 CO2 + GA |
| 111: | Gluext + 12 O = 6 CO2 |
| 112: | 2 Gluext + 10 O = 5 CO2 + GA |
| 113: | 2 Gluext + 10 O = 5 CO2 + GA |
| 114: | 2 Gluext + 11 O = 5 CO2 + GA |
| 115: | 1.625 Gluext + 4.20833 O = 2.75 CO2 + GA |
| 116: | 1.625 Gluext + 4.20833 O = 2.75 CO2 + GA |
| 117: | 2.66667 Gluext + 15.6667 O = 9 CO2 + GA |
| 118: | 2.66667 Gluext + 15.6667 O = 9 CO2 + GA |
| 119: | 5 Gluext + 41.3333 O = 23 CO2 + GA |
| 120: | 5 Gluext + 41.3333 O = 23 CO2 + GA |
| 121: | 1.375 Gluext + 2.45833 O = 1.25 CO2 + GA |
| 122: | 1.66667 Gluext + 5.66667 O = 3 CO2 + GA |
| 123: | 3 Gluext + 20.3333 O = 11 CO2 + GA |
| 124: | Gluext + 12 O = 6 CO2 |
| 125: | 1.88889 Gluext + 8.66667 O = 4.33333 CO2 + GA |
| 126: | 1.88889 Gluext + 8.66667 O = 4.33333 CO2 + GA |
| 127: | 1.5 Gluext + 4 O = 2 CO2 + GA |
| 128: | 1.5 Gluext + 4 O = 2 CO2 + GA |
| 129: | 1.66667 Gluext + 6 O = 3 CO2 + GA |
| 130: | 1.66667 Gluext + 6 O = 3 CO2 + GA |
| 131: | 2.5 Gluext + 4 O = CO2 + 2 GA |
| 132: | 1.5 Gluext + 5 O = 2 CO2 + GA |
| 133: | 1.65385 Gluext + 4.19231 O = 2.92308 CO2 + GA |
| 134: | 1.65385 Gluext + 4.19231 O = 2.92308 CO2 + GA |
| 135: | 1.61905 Gluext + 3.80952 O = 2.71429 CO2 + GA |
| 136: | 1.61905 Gluext + 3.80952 O = 2.71429 CO2 + GA |
| 137: | 1.38462 Gluext + 2.23077 O = 1.30769 CO2 + GA |
| 138: | 1.3619 Gluext + 1.98095 O = 1.17143 CO2 + GA |
| 139: | Gluext + 11 O = 6 CO2 |
| 140: | Gluext + 11 O = 6 CO2 |
| 141: | 1.70833 Gluext + 4.79167 O = 3.25 CO2 + GA |
| 142: | 1.70833 Gluext + 4.79167 O = 3.25 CO2 + GA |
| 143: | 1.68056 Gluext + 4.48611 O = 3.08333 CO2 + GA |
| 144: | 1.68056 Gluext + 4.48611 O = 3.08333 CO2 + GA |
| 145: | 1.63636 Gluext + 4 O = 2.81818 CO2 + GA |
| 146: | 1.63636 Gluext + 4 O = 2.81818 CO2 + GA |
| 147: | 1.7265 Gluext + 4.99145 O = 3.35897 CO2 + GA |
| 148: | 1.7265 Gluext + 4.99145 O = 3.35897 CO2 + GA |
| 149: | 1.87037 Gluext + 6.57407 O = 4.22222 CO2 + GA |
| 150: | 1.87037 Gluext + 6.57407 O = 4.22222 CO2 + GA |
| 151: | 1.45833 Gluext + 3.04167 O = 1.75 CO2 + GA |
| 152: | 1.38889 Gluext + 2.27778 O = 1.33333 CO2 + GA |
| 153: | 1.36364 Gluext + 2 O = 1.18182 CO2 + GA |
| 154: | 1.39316 Gluext + 2.32479 O = 1.35897 CO2 + GA |
| 155: | 1.50926 Gluext + 3.60185 O = 2.05556 CO2 + GA |
| 156: | Gluext + 11 O = 6 CO2 |
| 157: | Gluext + 11 O = 6 CO2 |
| 158: | 1.63636 Gluext + 4 O = 2.81818 CO2 + GA |
| 159: | 1.63636 Gluext + 4 O = 2.81818 CO2 + GA |
| 160: | 1.6875 Gluext + 4.5625 O = 3.125 CO2 + GA |
| 161: | 1.6875 Gluext + 4.5625 O = 3.125 CO2 + GA |
| 162: | 1.65385 Gluext + 4.19231 O = 2.92308 CO2 + GA |
| 163: | 1.65385 Gluext + 4.19231 O = 2.92308 CO2 + GA |
| 164: | 1.6875 Gluext + 4.5625 O = 3.125 CO2 + GA |
| 165: | 1.6875 Gluext + 4.5625 O = 3.125 CO2 + GA |
| 166: | 1.63636 Gluext + 4 O = 2.81818 CO2 + GA |
| 167: | 1.63636 Gluext + 4 O = 2.81818 CO2 + GA |
| 168: | 1.63636 Gluext + 4 O = 2.81818 CO2 + GA |
| 169: | 1.63636 Gluext + 4 O = 2.81818 CO2 + GA |
| 170: | 1.63636 Gluext + 4 O = 2.81818 CO2 + GA |
| 171: | 1.63636 Gluext + 4 O = 2.81818 CO2 + GA |
| 172: | 1.69231 Gluext + 4.61538 O = 3.15385 CO2 + GA |
| 173: | 1.69231 Gluext + 4.61538 O = 3.15385 CO2 + GA |
| 174: | 1.6875 Gluext + 4.5625 O = 3.125 CO2 + GA |
| 175: | 1.6875 Gluext + 4.5625 O = 3.125 CO2 + GA |
| 176: | 1.6875 Gluext + 4.5625 O = 3.125 CO2 + GA |
| 177: | 1.6875 Gluext + 4.5625 O = 3.125 CO2 + GA |
| 178: | 1.83333 Gluext + 6.16667 O = 4 CO2 + GA |
| 179: | 1.83333 Gluext + 6.16667 O = 4 CO2 + GA |
| 180: | 1.72222 Gluext + 4.94444 O = 3.33333 CO2 + GA |
| 181: | 1.72222 Gluext + 4.94444 O = 3.33333 CO2 + GA |
| 182: | 1.66667 Gluext + 4.33333 O = 3 CO2 + GA |
| 183: | 1.66667 Gluext + 4.33333 O = 3 CO2 + GA |
| 184: | 1.4 Gluext + 2.4 O = 1.4 CO2 + GA |
| 185: | 1.36364 Gluext + 2 O = 1.18182 CO2 + GA |
| 186: | 1.5 Gluext + 3.5 O = 2 CO2 + GA |
| 187: | 1.40625 Gluext + 2.46875 O = 1.4375 CO2 + GA |
| 188: | 1.5 Gluext + 3.5 O = 2 CO2 + GA |
| 189: | 1.40385 Gluext + 2.44231 O = 1.42308 CO2 + GA |
| 190: | 1.40625 Gluext + 2.46875 O = 1.4375 CO2 + GA |
| 191: | 1.4375 Gluext + 2.8125 O = 1.625 CO2 + GA |
| 192: | 1.39583 Gluext + 2.35417 O = 1.375 CO2 + GA |
| 193: | 1.36364 Gluext + 2 O = 1.18182 CO2 + GA |
| 194: | 1.36364 Gluext + 2 O = 1.18182 CO2 + GA |
| 195: | 1.35897 Gluext + 1.94872 O = 1.15385 CO2 + GA |
| 196: | 1.40625 Gluext + 2.46875 O = 1.4375 CO2 + GA |
| 197: | 1.47222 Gluext + 3.19444 O = 1.83333 CO2 + GA |
| 198: | 1.36667 Gluext + 2.03333 O = 1.2 CO2 + GA |
| 199: | 1.5 Gluext + 3 O = 2 CO2 + GA |
| 200: | 1.5 Gluext + 3 O = 2 CO2 + GA |
| 201: | 1.5 Gluext + 3.16667 O = 2 CO2 + GA |
| 202: | 1.5 Gluext + 3.16667 O = 2 CO2 + GA |
| 203: | 1.5 Gluext + 3.11538 O = 2 CO2 + GA |
| 204: | 1.5 Gluext + 3.11538 O = 2 CO2 + GA |
| 205: | 1.38462 Gluext + 2.15385 O = 1.30769 CO2 + GA |
| 206: | 1.38462 Gluext + 2.15385 O = 1.30769 CO2 + GA |
| 207: | 1.56522 Gluext + 3.65217 O = 2.3913 CO2 + GA |
| 208: | 1.56522 Gluext + 3.65217 O = 2.3913 CO2 + GA |
| 209: | Gluext + 11 O = 6 CO2 |
| 210: | Gluext + 11 O = 6 CO2 |
| 211: | 1.70833 Gluext + 4.79167 O = 3.25 CO2 + GA |
| 212: | 1.70833 Gluext + 4.79167 O = 3.25 CO2 + GA |
| 213: | 2.66667 Gluext + 15.3333 O = 9 CO2 + GA |
| 214: | 2.66667 Gluext + 15.3333 O = 9 CO2 + GA |
| 215: | 5.33333 Gluext + 44.6667 O = 25 CO2 + GA |
| 216: | 5.33333 Gluext + 44.6667 O = 25 CO2 + GA |
| 217: | 1.45833 Gluext + 3.04167 O = 1.75 CO2 + GA |
| 218: | 1.66667 Gluext + 5.33333 O = 3 CO2 + GA |
| 219: | 3.33333 Gluext + 23.6667 O = 13 CO2 + GA |
| 220: | Gluext + 11 O = 6 CO2 |
| 221: | Gluext + 11 O = 6 CO2 |
| 222: | 1.70833 Gluext + 4.79167 O = 3.25 CO2 + GA |
| 223: | 1.70833 Gluext + 4.79167 O = 3.25 CO2 + GA |
| 224: | 2.22222 Gluext + 10.4444 O = 6.33333 CO2 + GA |
| 225: | 2.22222 Gluext + 10.4444 O = 6.33333 CO2 + GA |
| 226: | 3.33333 Gluext + 22.6667 O = 13 CO2 + GA |
| 227: | 3.33333 Gluext + 22.6667 O = 13 CO2 + GA |
| 228: | 1.45833 Gluext + 3.04167 O = 1.75 CO2 + GA |
| 229: | 1.55556 Gluext + 4.11111 O = 2.33333 CO2 + GA |
| 230: | 2.33333 Gluext + 12.6667 O = 7 CO2 + GA |
| 231: | 2 Gluext + 8 O = 5 CO2 + GA |
| 232: | 2 Gluext + 8 O = 5 CO2 + GA |
| 233: | 3 Gluext + 19 O = 11 CO2 + GA |
| 234: | 3 Gluext + 19 O = 11 CO2 + GA |
| 235: | 2 Gluext + 8 O = 5 CO2 + GA |
| 236: | 2 Gluext + 8 O = 5 CO2 + GA |
| 237: | 1.5 Gluext + 3.5 O = 2 CO2 + GA |
| 238: | 1.5 Gluext + 3.5 O = 2 CO2 + GA |
| 239: | 1.4375 Gluext + 2.8125 O = 1.625 CO2 + GA |
| 240: | 2 Gluext + 9 O = 5 CO2 + GA |
| 241: | Gluext + 12 O = 6 CO2 |
| 242: | 1.62963 Gluext + 4.24074 O = 2.77778 CO2 + GA |
| 243: | 1.62963 Gluext + 4.24074 O = 2.77778 CO2 + GA |
| 244: | 1.66667 Gluext + 4.5 O = 3 CO2 + GA |
| 245: | 1.66667 Gluext + 4.5 O = 3 CO2 + GA |
| 246: | 1.49123 Gluext + 3.89474 O = 1.94737 CO2 + GA |
| 247: | 1.49123 Gluext + 3.89474 O = 1.94737 CO2 + GA |
| 248: | 1.5 Gluext + 4 O = 2 CO2 + GA |
| 249: | 1.5 Gluext + 4 O = 2 CO2 + GA |
| 250: | 1.5303 Gluext + 4.36364 O = 2.18182 CO2 + GA |
| 251: | 1.5303 Gluext + 4.36364 O = 2.18182 CO2 + GA |
| 252: | 1.35185 Gluext + 2.2963 O = 1.11111 CO2 + GA |
| 253: | 1.35417 Gluext + 2.3125 O = 1.125 CO2 + GA |
| 254: | 2.38333 Gluext + 3.9 O = CO2 + 1.9 GA |
| 255: | 2.5 Gluext + 4 O = CO2 + 2 GA |
| 256: | 1.33333 Gluext + 2 O = 1 CO2 + GA |
| 257: | 1.63768 Gluext + 2.55072 O = CO2 + 1.26087 GA |
| 258: | 1.72222 Gluext + 2.66667 O = CO2 + 1.33333 GA |
| 259: | 1.33333 Gluext + 2.25 O = CO2 + GA |
| 260: | 1.86364 Gluext + 3.08333 O = CO2 + 1.45455 GA |
| 261: | 1.98148 Gluext + 3.22222 O = CO2 + 1.55556 GA |
| 262: | 1.33333 Gluext + 2.66667 O = CO2 + 1 GA |
| 263: | 1.33333 Gluext + 2.66667 O = CO2 + 1 GA |
| 264: | Gluext + 12 O = 6 CO2 |
| 265: | 1.5 Gluext + 4 O = 2 CO2 + GA |
| 266: | 1.5 Gluext + 4 O = 2 CO2 + GA |
| 267: | 1.5 Gluext + 4 O = 2 CO2 + GA |
| 268: | 1.5 Gluext + 4 O = 2 CO2 + GA |
| 269: | 1.64286 Gluext + 4.42857 O = 2.85714 CO2 + GA |
| 270: | 1.64286 Gluext + 4.42857 O = 2.85714 CO2 + GA |
| 271: | 2.85 Gluext + 4.3 O = CO2 + 2.3 GA |
| 272: | 2.5 Gluext + 4 O = CO2 + 2 GA |
| 273: | 1.5 Gluext + 5 O = 2 CO2 + GA |
| 274: | 1.375 Gluext + 2.375 O = 1.25 CO2 + GA |
| 275: | 2.5 Gluext + 4 O = CO2 + 2 GA |
| 276: | 1.625 Gluext + 2.5 O = CO2 + 1.25 GA |
| 277: | 1.86364 Gluext + 3.04545 O = CO2 + 1.45455 GA |
| 278: | 1.42308 Gluext + 2.38462 O = CO2 + 1.07692 GA |
| 279: | Gluext + 12 O = 6 CO2 |
| 280: | Gluext + 12 O = 6 CO2 |
| 281: | 1.77778 Gluext + 7.33333 O = 3.66667 CO2 + GA |
| 282: | 1.77778 Gluext + 7.33333 O = 3.66667 CO2 + GA |
| 283: | 1.66667 Gluext + 6 O = 3 CO2 + GA |
| 284: | 1.66667 Gluext + 6 O = 3 CO2 + GA |
| 285: | 1.33333 Gluext + 3 O = CO2 + GA |
| 286: | 1.33333 Gluext + 2.33333 O = CO2 + 1 GA |
| 287: | 1.33333 Gluext + 2.66667 O = CO2 + 1 GA |
| 288: | 1.5 Gluext + 5 O = 2 CO2 + GA |

**Supplementary Table S3**: List of elementary modes with the highest maximum yields and the fluxes in each reaction that compose them, according to the output file generated by Metatool. AD (1-2): Reactions that turn Pyruvate (PIR) into 2-Phosphoenolpyruvate (PEP); AS (1-7): Reactions from Shikimic Acid Pathway; CK (1-8): Reactions from the Citric Acid Cycle; ED (1-2): Reactions from the Entner-Doudoroff pathway; EMP (1-10): Reactions from the Embden-Meyerhof Parnas pathway; GA_SINT (1-6): Possible reactions included for gallic acid production using intermediates from the shikimic acid pathway. GA_SINT1 and 2 are the reactions that make Route 1, GA_SINT3, 5 and 2 make Route 2 and GA_SINT4, 6 and 2 make Route 3; GLN3: Single reaction that turns Fructose 1,6-bisphosphate (F16P) into F6P; GLY (1-2): Reactions from the acquisition and transformation of external Glycerol (Glyext) into Fructose-6-phosphate (F6P); OXFAD and OXNAD: Reactions that represent the oxidation of FADH and NADH, respectively; PR (1-5): Reactions that represent peripheral catalysis of Glucose (Gluext) into 6-Phosphogluconate (PG6); STHA_PNTAB: Reactions catalyzed by the transhydrogenases PntAB or SthA; VP (1-10): Reactions from the Pentose Phosphate Pathway:

| **Carbon source** | **Synthetic Route for GA production** | **Elementary mode** | **Reactions and metabolic fluxes** |
| --- | --- | --- | --- |
| Glycerol | Route 1 | 54 | (31) (1.26667 GLN3) (0.2 EMP10) (9.06667 GLY) (3.8 AS1) (3.8 AS2) (3.8 GA_SINT1) (3.8 GA_SINT2) (0.2 CK1) (0.2 CK2) (0.2 CK3) (0.2 CK4) (0.2 CK5) (0.2 CK6) (0.2 CK7) (0.2 CK8) (0.2 CPD) OXNAD (0.2 OXFAD) (-1.26667 EMP4) (7.8 EMP5) (4 EMP6) (4 EMP7) (4 EMP8) (4 EMP9) (1.26667 VP6) (-1.26667 VP7) (1.26667 VP8) (1.26667 VP9) (-2.53333 VP10) (3.8 AS3) (-3.6 STHA_PNTAB) irreversible |
| Glycerol | Route 3 | 4 | (37) (0.333333 GLN3) (2.83333 GLY) AS1 AS2 GA_SINT2 AS5 AS7 (0.5 CK1) (0.5 CK2) (0.5 CK3) (0.5 CK4) (0.5 CK5) (0.5 CK6) (0.5 CK7) (0.5 CK8) (0.5 CPD) (0.5 OXNAD) (0.5 OXFAD) (0.5 AD1) (0.5 AD2) (-0.333333 EMP4) (2.5 EMP5) (1.5 EMP6) (1.5 EMP7) (1.5 EMP8) (1.5 EMP9) (0.333333 VP6) (-0.333333 VP7) (0.333333 VP8) (0.333333 VP9) (-0.666667 VP10) AS3 AS4 AS6 GA_SINT4 GA_SINT6 (-2.5 STHA_PNTAB) irreversible |
| Glycerol | Route 2 | 5 | (37) (0.266667 GLN3) (2.26667 GLY) (0.8 AS1) (0.8 AS2) (0.8 GA_SINT2) (0.8 AS5) (0.8 AS7) (0.4 CK1) (0.4 CK2) (0.4 CK3) (0.4 CK4) (0.4 CK5) (0.4 CK6) (0.4 CK7) (0.4 CK8) (0.4 CPD) (0.4 OXNAD) (0.4 OXFAD) (0.4 AD1) (0.4 AD2) (-0.266667 EMP4) (2 EMP5) (1.2 EMP6) (1.2 EMP7) (1.2 EMP8) (1.2 EMP9) (0.266667 VP6) (-0.266667 VP7) (0.266667 VP8) (0.266667 VP9) (-0.533333 VP10) (0.8 AS3) (0.8 AS4) (0.8 AS6) (0.8 GA_SINT3) (0.8 GA_SINT5) (-2 STHA_PNTAB) irreversible |
| Glucose | Route 1 | 76 | (33) EMP1 (0.730496 ED1) (0.730496 ED2) (0.730496 VP1) (0.808511 AS1) (0.808511 AS2) (0.808511 GA_SINT1) (0.808511 GA_SINT2) (0.113475 CK1) (0.113475 CK2) (0.113475 CK3) (0.113475 CK4) (0.113475 CK5) (0.113475 CK6) (0.113475 CK7) (0.113475 CK8) (0.113475 CPD) (0.567376 OXNAD) (0.113475 OXFAD) (0.617021 AD1) (0.617021 AD2) (0.269504 EMP2) (0.191489 EMP6) (0.191489 EMP7) (0.191489 EMP8) (0.191489 EMP9) (0.269504 VP6) (-0.269504 VP7) (0.269504 VP8) (0.269504 VP9) (-0.539007 VP10) (0.808511 AS3) (0.035461 STHA_PNTAB) irreversible |
| Glucose | Route 1 | 98 | (24) EMP1 (0.617021 ED1) (0.617021 ED2) (0.957447 VP1) (0.340426 VP5) (0.808511 AS1) (0.808511 AS2) (0.808511 GA_SINT1) (0.808511 GA_SINT2) (0.680851 OXNAD) (0.617021 AD1) (0.617021 AD2) (0.0425532 EMP2) (0.191489 EMP6) (0.191489 EMP7) (0.191489 EMP8) (0.191489 EMP9) (0.382979 VP6) (-0.0425532 VP7) (0.382979 VP8) (0.382979 VP9) (-0.425532 VP10) (0.808511 AS3) (0.489362 STHA_PNTAB) irreversible |
| Glucose | Route 3 | 73 | (38) (1.47368 EMP1) (1.14035 ED1) (1.14035 ED2) (1.14035 VP1) AS1 AS2 GA_SINT2 AS5 AS7 (0.614035 CK1) (0.614035 CK2) (0.614035 CK3) (0.614035 CK4) (0.614035 CK5) (0.614035 CK6) (0.614035 CK7) (0.614035 CK8) (0.614035 CPD) (1.07018 OXNAD) (0.614035 OXFAD) (1.52632 AD1) (1.52632 AD2) (0.333333 EMP2) (0.473684 EMP6) (0.473684 EMP7) (0.473684 EMP8) (0.473684 EMP9) (0.333333 VP6) (-0.333333 VP7) (0.333333 VP8) (0.333333 VP9) (-0.666667 VP10) AS3 AS4 AS6 GA_SINT4 GA_SINT6 (-1.24561 STHA_PNTAB) irreversible |
| Glucose | Route 2 | 74 | 74: (38) (0.965517 EMP1) (0.747126 ED1) (0.747126 ED2) (0.747126 VP1) (0.655172 AS1) (0.655172 AS2) (0.655172 GA_SINT2) (0.655172 AS5) (0.655172 AS7) (0.402299 CK1) (0.402299 CK2) (0.402299 CK3) (0.402299 CK4) (0.402299 CK5) (0.402299 CK6) (0.402299 CK7) (0.402299 CK8) (0.402299 CPD) (0.701149 OXNAD) (0.402299 OXFAD) AD1 AD2 (0.218391 EMP2) (0.310345 EMP6) (0.310345 EMP7) (0.310345 EMP8) (0.310345 EMP9) (0.218391 VP6) (-0.218391 VP7) (0.218391 VP8) (0.218391 VP9) (-0.436782 VP10) (0.655172 AS3) (0.655172 AS4) (0.655172 AS6) (0.655172 GA_SINT3) (0.655172 GA_SINT5) (-0.816092 STHA_PNTAB) irreversible |
| Glucose | Route 3 | 81 | (29) (1.47368 EMP1) (0.526316 ED1) (0.526316 ED2) (2.36842 VP1) (1.84211 VP5) AS1 AS2 GA_SINT2 AS5 AS7 (1.68421 OXNAD) (1.52632 AD1) (1.52632 AD2) (-0.894737 EMP2) (0.473684 EMP6) (0.473684 EMP7) (0.473684 EMP8) (0.473684 EMP9) (0.947368 VP6) (0.894737 VP7) (0.947368 VP8) (0.947368 VP9) (-0.0526316 VP10) AS3 AS4 AS6 GA_SINT4 GA_SINT6 (1.21053 STHA_PNTAB) irreversible |
| Glucose | Route 2 | 82 | (29) (0.622222 EMP1) (0.222222 ED1) (0.222222 ED2) VP1 (0.777778 VP5) (0.422222 AS1) (0.422222 AS2) (0.422222 GA_SINT2) (0.422222 AS5) (0.422222 AS7) (0.711111 OXNAD) (0.644444 AD1) (0.644444 AD2) (-0.377778 EMP2) (0.2 EMP6) (0.2 EMP7) (0.2 EMP8) (0.2 EMP9) (0.4 VP6) (0.377778 VP7) (0.4 VP8) (0.4 VP9) (-0.0222222 VP10) (0.422222 AS3) (0.422222 AS4) (0.422222 AS6) (0.422222 GA_SINT3) (0.422222 GA_SINT5) (0.511111 STHA_PNTAB) irreversible |
| Glucose | Route 3 | 91 | (37) (6.06811 EMP1) (4.00929 ED1) (4.00929 ED2) (6.06811 VP1) (2.05882 VP5) (4.11765 AS1) (4.11765 AS2) (4.11765 GA_SINT2) (4.11765 AS5) (4.11765 AS7) (1.84211 CK1) (1.84211 CK2) (1.84211 CK3) (1.84211 CK4) (1.84211 CK5) (1.84211 CK6) (1.84211 CK7) (1.84211 CK8) (1.84211 CPD) (5.09288 OXNAD) (1.84211 OXFAD) (6.28483 AD1) (6.28483 AD2) (1.95046 EMP6) (1.95046 EMP7) (1.95046 EMP8) (1.95046 EMP9) (2.05882 VP6) (2.05882 VP8) (2.05882 VP9) (-2.05882 VP10) (4.11765 AS3) (4.11765 AS4) (4.11765 AS6) (4.11765 GA_SINT4) (4.11765 GA_SINT6) (-2.3839 STHA_PNTAB) irreversible |
| Glucose | Route 2 | 92 | (37) (6.06811 EMP1) (4.00929 ED1) (4.00929 ED2) (6.06811 VP1) (2.05882 VP5) (4.11765 AS1) (4.11765 AS2) (4.11765 GA_SINT2) (4.11765 AS5) (4.11765 AS7) (1.84211 CK1) (1.84211 CK2) (1.84211 CK3) (1.84211 CK4) (1.84211 CK5) (1.84211 CK6) (1.84211 CK7) (1.84211 CK8) (1.84211 CPD) (5.09288 OXNAD) (1.84211 OXFAD) (6.28483 AD1) (6.28483 AD2) (1.95046 EMP6) (1.95046 EMP7) (1.95046 EMP8) (1.95046 EMP9) (2.05882 VP6) (2.05882 VP8) (2.05882 VP9) (-2.05882 VP10) (4.11765 AS3) (4.11765 AS4) (4.11765 AS6) (4.11765 GA_SINT3) (4.11765 GA_SINT5) (-2.3839 STHA_PNTAB) irreversible |
| Glucose | Route 3 | 93 | (38) (3.76285 EMP1) (2.1166 ED1) (2.1166 ED2) (4.50198 VP1) (2.38538 VP5) (2.55336 AS1) (2.55336 AS2) (2.55336 GA_SINT2) (2.55336 AS5) (2.55336 AS7) (0.772727 CK1) (0.772727 CK2) (0.772727 CK3) (0.772727 CK4) (0.772727 CK5) (0.772727 CK6) (0.772727 CK7) (0.772727 CK8) (0.772727 CPD) (3.52767 OXNAD) (0.772727 OXFAD) (3.89723 AD1) (3.89723 AD2) (-0.73913 EMP2) (1.20949 EMP6) (1.20949 EMP7) (1.20949 EMP8) (1.20949 EMP9) (1.64625 VP6) (0.73913 VP7) (1.64625 VP8) (1.64625 VP9) (-0.907115 VP10) (2.55336 AS3) (2.55336 AS4) (2.55336 AS6) (2.55336 GA_SINT4) (2.55336 GA_SINT6) irreversible |
| Glucose | Route 2 | 94 | (38) (2.40049 EMP1) (1.35028 ED1) (1.35028 ED2) (2.87201 VP1) (1.52174 VP5) (1.6289 AS1) (1.6289 AS2) (1.6289 GA_SINT2) (1.6289 AS5) (1.6289 AS7) (0.492958 CK1) (0.492958 CK2) (0.492958 CK3) (0.492958 CK4) (0.492958 CK5) (0.492958 CK6) (0.492958 CK7) (0.492958 CK8) (0.492958 CPD) (2.25046 OXNAD) (0.492958 OXFAD) (2.48622 AD1) (2.48622 AD2) (-0.471525 EMP2) (0.771586 EMP6) (0.771586 EMP7) (0.771586 EMP8) (0.771586 EMP9) (1.05021 VP6) (0.471525 VP7) (1.05021 VP8) (1.05021 VP9) (-0.57869 VP10) (1.6289 AS3) (1.6289 AS4) (1.6289 AS6) (1.6289 GA_SINT3) (1.6289 GA_SINT5) irreversible |

**Supplementary Table S4**: List of all primers used in this work:

| **Primers used to amplify and clone genes** | *qui*C fwd | CCCATAGAATTCAGGAGGAAAAACATATGAATACATTACGTTTAACTACTCTTGCATTAGG |
| --- | --- | --- |
|  | *qui*C rev | TCCTTACCCGGGTTATTTAGTTTGTACGGCCGAACTAAGTTCAGAC |
|  | *pob*A* fwd | CCATAACCCGGGAGGAGGAAAAACATATGAAGACTCAAGTCGCCATCATCGGCGC |
|  | *pob*A* rev | CGGCGTACTAGTTCATTCGACGGCCTCGTAGGGCAGTCCCACGAAGTTCTCG |
|  | *aro*G4 H1 fwd | CCTGCAGGTCGACTCTAGAGACTAGTAGGAGGAAAAACATATGAATTATCAGAACGACG |
|  | *aro*G4 H1 rev | CGAGATATTGTAGGGTGATCATATCGAG |
|  | *aro*G4 H2 fwd | GATCACCCTACAATATCTCGCTGACCTGATGAGC |
|  | *aro*G4 H2 rev | ATTCGAGCTCGGTACCCGGGGAGCTCTTACCCGCGACGCGCTTT |
| **Primers used to amplify the sequences that formed the recombination templates** | *gal*TAPR H1 fwd | CCTGCAGGTCGACTCTAGAGTCATGCCAGGTTCTCCGTC |
|  | *gal*TAPR H1 rev | ACCACGCATGGATCATAAGGGCTCCCGG |
|  | *gal*TAPR H2 fwd | CCTTATGATCCATGCGTGGTCACCTTTG |
|  | *gal*TAPR H2 rev | ATTCGAGCTCGGTACCCGGGATCAGGTACGCCATGGCAG |
|  | *pca*HG H1 fwd | CCTGCAGGTCGACTCTAGAGGGAAGTGCCATGGGTGAAG |
|  | *pca*HG H1 rev | CTTCACCTCACATGCCGGTTTCCTCTCTTG |
|  | *pca*HG H2 fwd | AACCGGCATGTGAGGTGAAGCTTGGGGC |
|  | *pca*HG H2 rev | ATTCGAGCTCGGTACCCGGGACGATTTCCCCATTGCCAG |
| **Primers used to validate the deletions through PCR** | *gal*TAPR test fwd | GAAGATTCTAGAATAGTGGATCAGCCCACGCC |
|  | *gal*TAPR test rev | GCCTTAGAATTCCCCGGCGCAATTCACACC |
|  | *pca*HG test fwd | GGTCTCACTTGGTCCCTGTT |
|  | *pca*HG test rev | CGAGAAGCACAAGGCAATCCT |
| **Primers used to sequence the plasmid JAQP** | JQP_f1 | CGTCACACTTTGCTATGC |
|  | JQP_f2 | ATCGCTTCTGTATTGGGG |
|  | JQP_f3 | TGGTTCATGTGTACCAGATC |
|  | JQP_f4 | ACAACGTGATCCTCGAAC |
|  | JQP_f5 | TTCGCCCTGTGCAGC |
|  | JQP_r1 | CGTTGTAAAACGACGGCC |
|  | JQP_r2 | TCGTCCGACCAGTCCTC |
|  | JQP_r3 | TGTTCCAGCACCCCG |
|  | JQP_r4 | TGCAGGCACAGCAAATACA |
|  | JQP_r5 | AAGGTTATTGCTTGAACGG |
|  | AroG Fwd1 | CCTGACGCTTTTTATCGCAAC |
|  | AroG Fwd2 | GTGAGTTTCTCGATATGATCACC |
|  | AroG Fwd3 | GAGCCTCGAGAGCGGGGA |
|  | AroG Rev1 | GGCTTGTTCAATTGCCCA |
|  | AroG Rev2 | CGCGCAGAATGATATGGC |
|  | AroG Rev3 | CCGCGACAGGATCATGAATT |
| **Primers used to sequence the deleted regions** | galTAPR 1 | ACAAAGTCGGCGGAATGTGC |
|  | galTAPR test rev | GCCTTAGAATTCCCCGGCGCAATTCACACC |
|  | PD 1 | CATCGACCACGTTGTCGCT |
|  | PD 2 | GCAACCCGGAAACGCTGG |


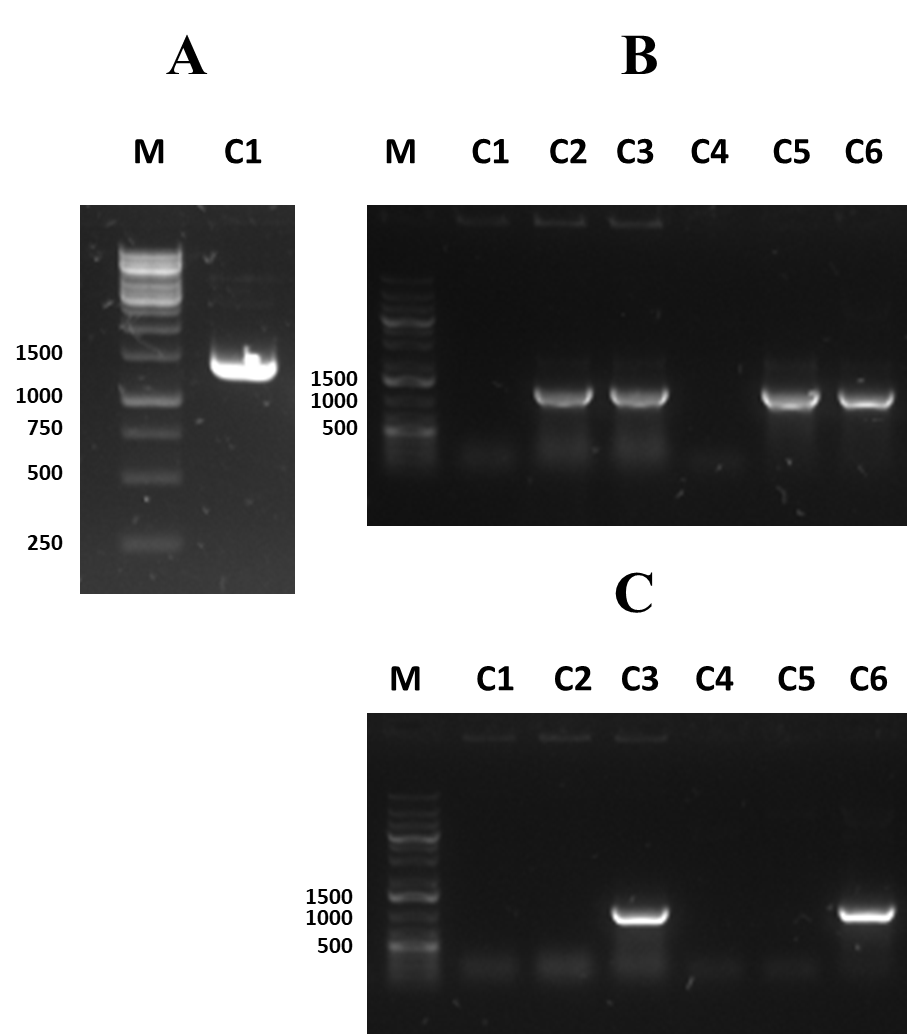


**Supplementary Figure S1**. Electrophoresis in 0.7% agarose gel performed to validate the insertions of *qui*C (A), *pob*A* (B) and *aro*G4 (C) in plasmid pJN105. The samples used were amplifications of DNA obtained directly from colonies and the primers were the same primers used to amplify those genes from bacterial genome (Supplementary Table S4). M: Molecular weight marker 1Kb Plus^tm^ (GoldBio) for (A) and GeneRuler 1 kb Plus DNA Ladder (Thermo Fischer^TM^) for (B) and (C); C1-6: Colonies tested.


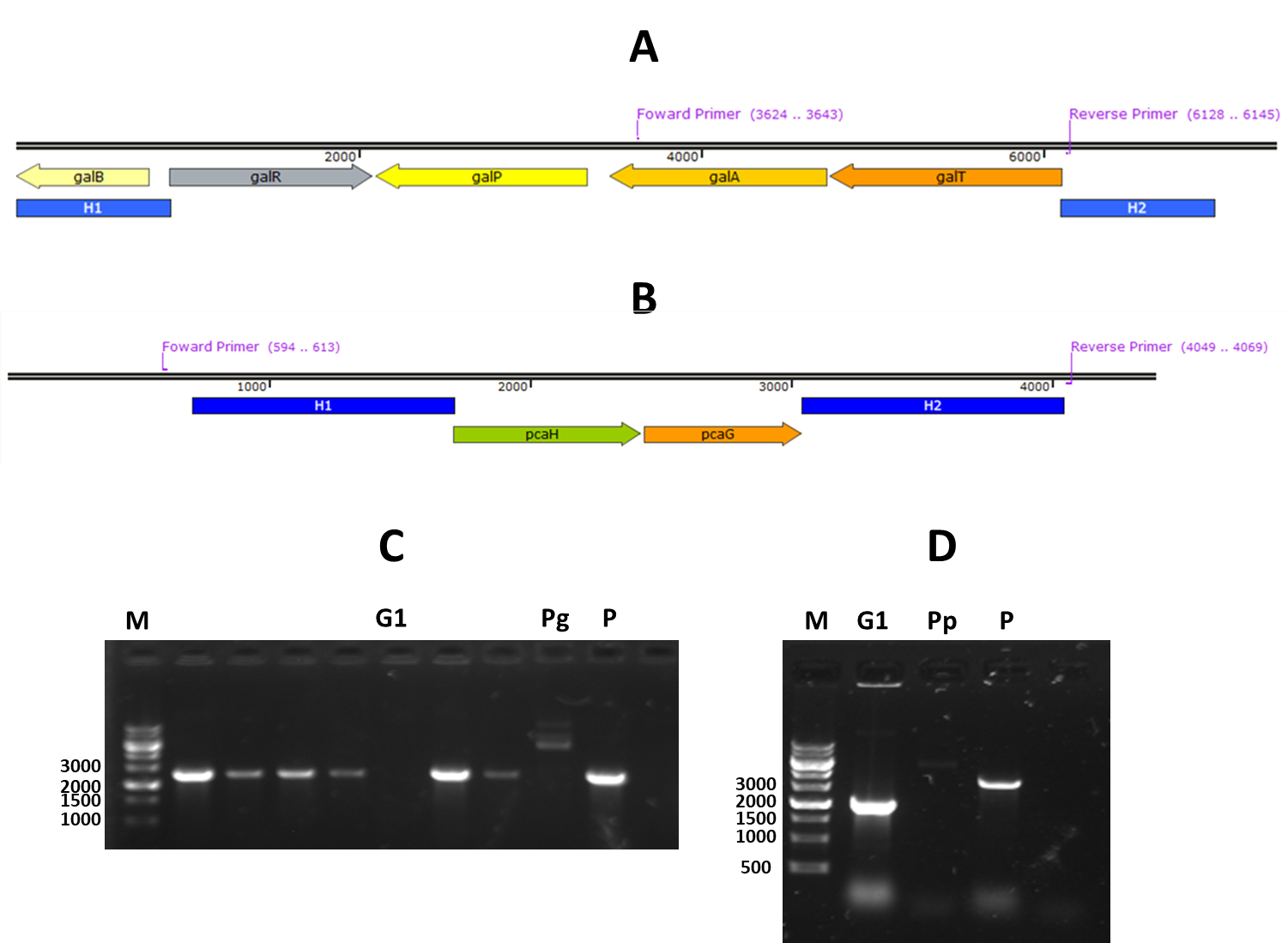


**Supplementary Figure S2**. (A) General scheme of the deleted cluster *gal*TAPR, flanked by the upstream and downstream homologous sequences H1 and H2 that formed the cassette for recombination (done with SnapGene: https://www.snapgene.com/); (B) General scheme of the deleted cluster *pca*HG, flanked by the upstream and downstream homologous sequences H1 and H2 that formed the cassette for recombination (done with SnapGene: https://www.snapgene.com/); (C and D) Electrophoresis in 0.7% agarose gel performed to validate the deletions of *gal*TAPR and *pca*HG, respectively, in the Gal1 strain. The samples used were amplifications of DNA obtained directly from colonies. M: Molecular marker 1Kb Ladder M1181 (Sinapse), G1: Genomic DNA from the Gal1 strain; Pg: Plasmid pEX18:: galTAPR cassette; Pp: Plasmid pEX18::*pcaHG cassette*; P: Genomic DNA from the parental strain. Once the primers used in both cases do not have homology with the cassette, the bands present in the wells referring to the plasmids can be considered nonspecific bands.

**
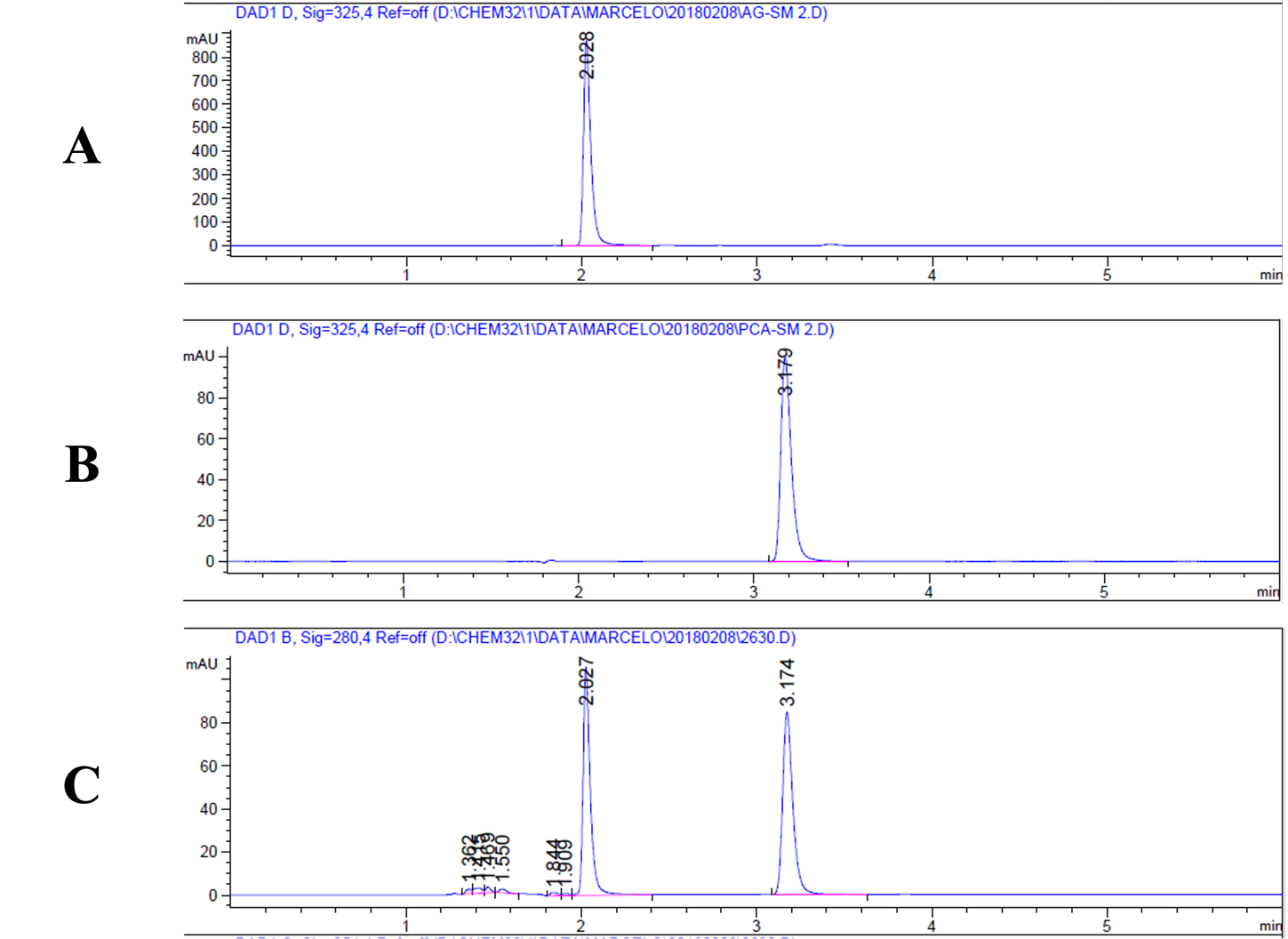
**

**Supplementary Figure S3.** Chromatogram of the PCA and GA production assay in P. putida Gal1 (2^nd^ assay). (A) Retention time of GA used as standard. (B) Retention time of PCA used as standard. (C) Chromatogram of a sample obtained from 72h culture of Gal1_JAQP.


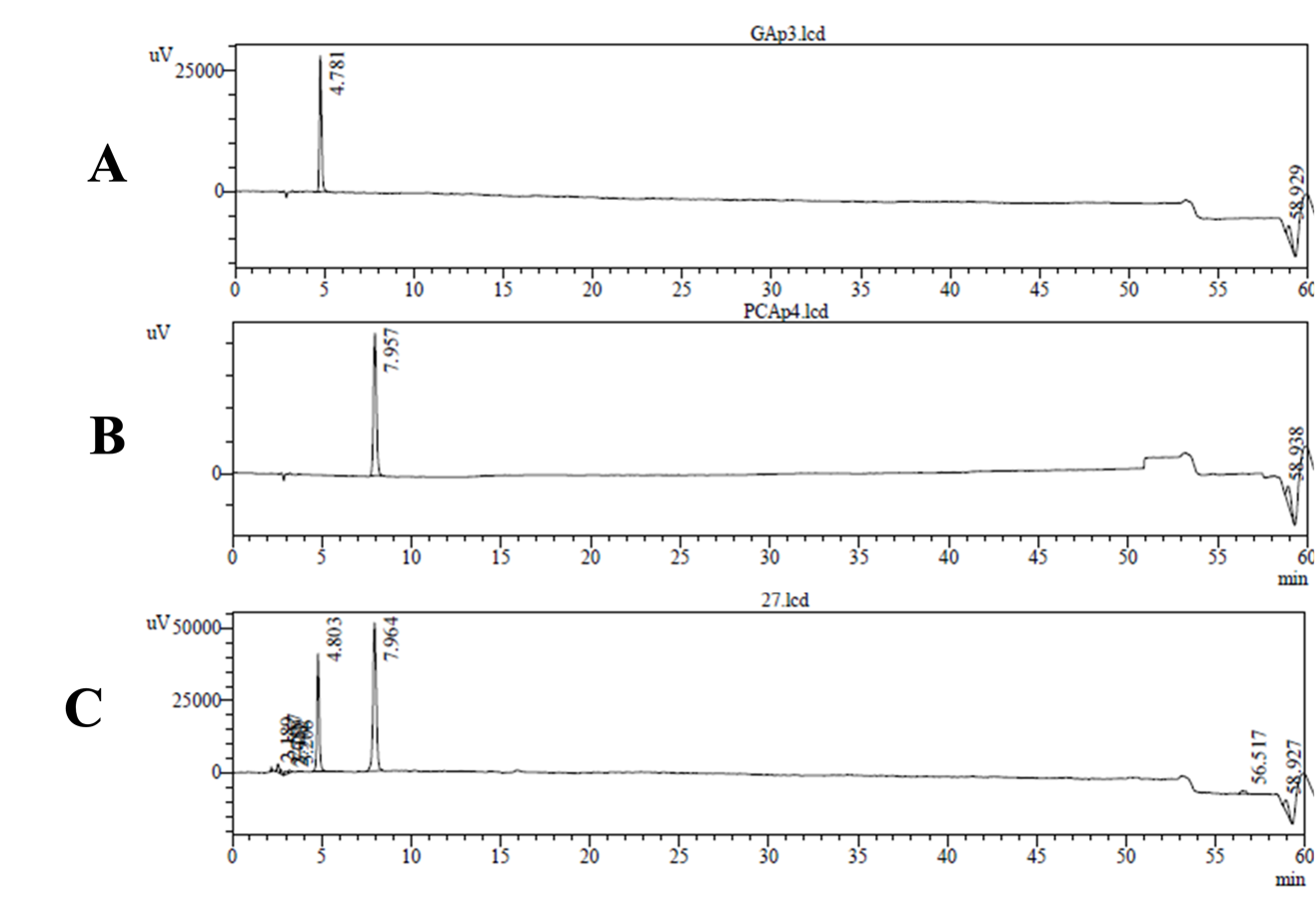


**Supplementary Figure S4.** Chromatogram of the PCA and GA production assay in P. putida Gal1 (3^rd^ assay). (A) Retention time of GA used as standard. (B) Retention time of PCA used as standard. (C) Chromatogram of a sample obtained from 72h culture of Gal1_JAQP.


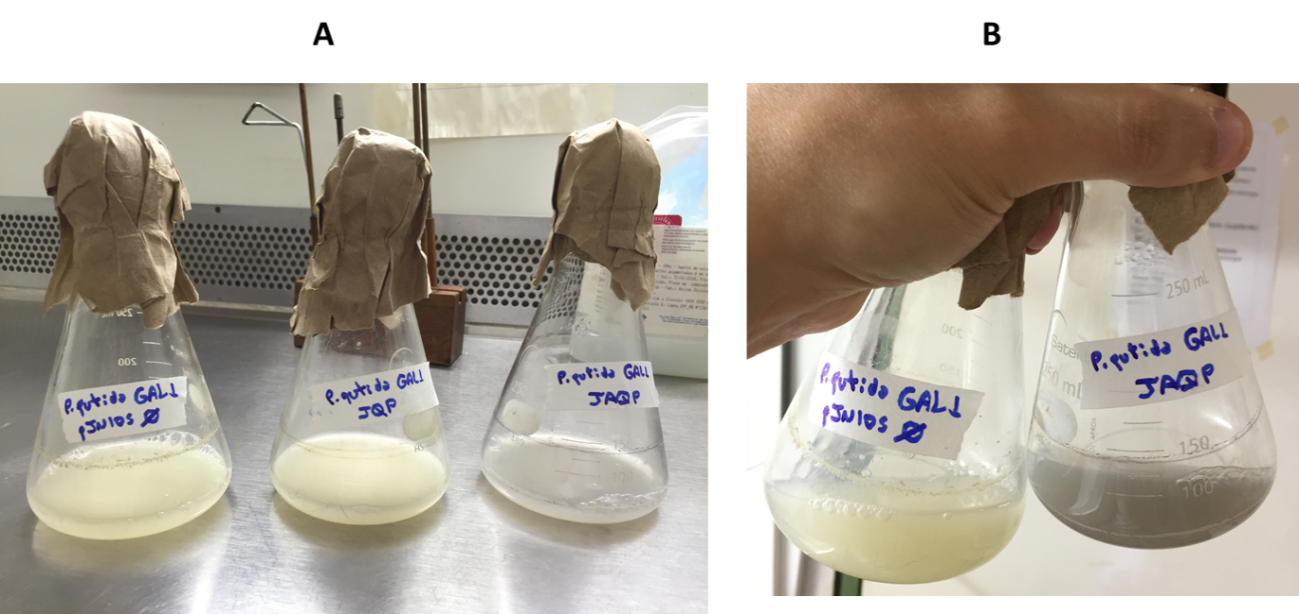


**Supplementary Figure S5.** Oxidation of the culture medium when Gal1 was cultivated with plasmid JAQP. A) Comparison of cultures with Gal1 transformed with J0 (control), JQP and JAQP. B) Closer look of the color difference between the medium with Gal1_J0 and Gal1_JAQP.
